# General Trends of the *Camelidae* Antibody V_H_Hs Domain Dynamics

**DOI:** 10.3390/ijms24054511

**Published:** 2023-02-24

**Authors:** Akhila Melarkode Vattekatte, Julien Diharce, Joseph Rebehmed, Frédéric Cadet, Fabrice Gardebien, Catherine Etchebest, Alexandre G. de Brevern

**Affiliations:** 1 Université de Paris, INSERM, BIGR, F-75014 Paris, France; 2 Université de la Réunion, INSERM, BIGR, F-97715 Saint-Denis, France; 3 Department of Computer Science and Mathematics, Lebanese American University, Beirut 1102 2801, Lebanon; 4 Artificial Intelligence Department, PEACCEL, Square Albin Cachot, F-75013 Paris, France

**Keywords:** molecular dynamics simulation, flexibility, mobility, disorder, structural alphabet, Protein Blocks, nanobody, single-chain antibody, sybody, antibody

## Abstract

Conformational flexibility plays an essential role in antibodies’ functional and structural stability. They facilitate and determine the strength of antigen–antibody interactions. Camelidae express an interesting subtype of single-chain antibody, named Heavy Chain only Antibody. They have only one N-terminal Variable domain (V_H_H) per chain, composed of Frameworks (FRs) and Complementarity Determining regions (CDRs) like their VH and VL counterparts in IgG. Even when expressed independently, V_H_H domains display excellent solubility and (thermo)stability, which helps them to retain their impressive interaction capabilities. Sequence and structural features of V_H_H domains contributing to these abilities have already been studied compared to classical antibodies. To have the broadest view and understand the changes in dynamics of these macromolecules, large-scale molecular dynamics simulations for a large number of non-redundant V_H_H structures have been performed for the first time. This analysis reveals the most prevalent movements in these domains. It reveals the four main classes of V_H_Hs dynamics. Diverse local changes were observed in CDRs with various intensities. Similarly, different types of constraints were observed in CDRs, while FRs close to CDRs were sometimes primarily impacted. This study sheds light on the changes in flexibility in different regions of V_H_H that may impact their in silico design.

## 1. Introduction

Antibodies (Abs) are the basis of the immune system in many species. Classical antibodies, such as Immunoglobulin Gamma (IgGs), are large macromolecular molecules composed of two chains forming a heterodimer. The IgG comprises: (i) a heavy chain with four distinct domains and (ii) a light chain with two distinct domains. At their N-terminus, a V_H_ domain (for heavy chain) and V_L_ domain (for light chain) are the binding sites to the epitope. Single-chain immunoglobulin is found in vertebrate species such as the nurse shark and the camelids. The latter is composed of genera of the ancient world of *Camelus* (Bactrian camel, dromedary camel) and from the new world of *Llama* (guanaco, llama) and *Vicugna* (alpaca, vicuña). They all have—in addition to IgGs—Heavy Chain Only Antibody (HCAb). HCAbs lack the light chain and have a smaller heavy chain with only one V_H_ (named then V_H_H and sometimes, for commercial purposes, Nanobody). Individually expressed V_H_H domains retain their ability to bind their epitope efficiently as classical antibodies. As they are small in size (<150 residues), they are being used in bio-therapeutics, e.g., against acquired thrombotic thrombocytopenic purpura [1], against rheumatoid arthritis [2,3], and recently against SARS-CoV-2 with variable potencies [4,5,6,7,8,9,10,11,12,13,14].

V_H_/V_L_ domains dictate antibody binding for IgGs, as do V_H_H for HCAbs. They all have interspersed amino acid regions with varying sequence conservation called Framework Regions (FRs) and Complementarity Determining Regions (CDRs). The former is expected to be preserved in sequence and structure, forming a very characteristic structural base; they are often denoted as FR1 to FR4. The three CDRs (CDR1 to CDR3) are interspersed with the FRs. Mostly the CDRs contact the epitope and are particularly variable in sequence and conformation, contributing to the specificity of each antibody [15].

V_H_H domains have gathered an enormous interest in the antibody community with an impressive number of patents [16] and an extraordinary increase in deposited structures in the Protein Data Bank (PDB) [17] in the last years (more than 200 X-ray structures in 3 years) [18].

Due to the above reasons, investigating pertinent features of V_H_Hs would be very valuable. For instance, we published one of the first studies on the conformational diversity of FRs. It emphasised that even FRs show conformational diversification [19]. Similarly, we explored the sequence–structure relationship of V_H_Hs, which is not composed of successively conserved Framework Regions (FRs) and hypervariable (CDRs) regions but something more complicated [20]. These results explain why despite their supposed simplicity, it is not unchallenging to propose a relevant structural model of a given V_H_H domain [21].

The above analyses provide insights into conformational diversity in 3D structures and models but also suffer from apparent limitations. The most obvious is that only one conformation is considered in the conformational landscape. Hence, it is difficult to ascertain how conformational diversity in one region influences another similar region. For instance, in the case of the V_H_H domain, how do FR/CDRs influence other FRs/CDRs, or FRs influence conformational diversity in CDRs? Molecular Dynamics (MD) simulations are a perfect tool to apprehend the dynamics of these specific domains, and so to understand various macromolecular phenomena.

Using MDs to understand the stability of V_H_H domains has increased significantly in the past decade employing several kinds of MD techniques. Early studies on unbinding mechanics of a camelid V_H_H and its lysozyme target were carried out using steered molecular dynamics [22,23]. Replica exchange MD was used to understand the influence of multiple amino acid substitutions in hypervariable loop regions of a Llama V_H_H [24]. Classical MDs at two different temperatures were used to understand the influence of amino acid substitutions and V_H_H yield in experimental conditions [25]. Investigations of V_H_H domain thermostability were assessed using classical MD at eight different temperatures for a specific V_H_H by analysing the conservation of native contacts and changes in flexibility for in FRs and CDRs [26].

In contrast, in another study, seven different V_H_Hs were analysed using classical MDs at three different temperatures [27]. The latter looked at the improvement in thermostability; their main observation about the change of CDR1 residues was experimentally validated [28]. Another study showed that MD could efficiently evaluate binding affinity (modelled and docked) from V_H_H-target simulations [29]. A recent study has explored the differences in stability of V_H_H mutants and assessed the conformational space between two V_H_Hs, which differ only by nine amino acids [30]. Recently, Fernandez-Quintero and collaborators showed an excellent agreement of (i) NOE-derived distance maps obtained from NMR and (ii) MD simulations for an anti-GFP-binding V_H_H; they observed similar conformational spaces for the simulations [31].

Classical MD simulations were performed on V_H_H domains in complex with HIV capsid protein p24, and binding energy calculations from these MD studies helped the researchers identify key interfacial residues [32]. Another study used MD simulations of the stably expressed V_H_H proteins from phage libraries to ascertain whether the V_H_H clones used in the study possessed the required diverse CDR3 confirmations [33]. MD simulations were also used to assess chemical-induced V_H_H dimerisation to generate bivalent domains for biotechnological applications [34]. All these studies shed light on individual V_H_H dynamics using classical or advanced MDs.

This study aims further to simulate 88 non-redundant V_H_H domains at longer time scales using classical MD. The classical approaches, such as Root Mean Square Fluctuations (RMSFs), were used to analyse the different regions of V_H_Hs characterising different behaviours of V_H_Hs ranging from rigid to flexible. Additionally, an in-house developed method, structural alphabet (Protein Blocks) [35] offers a unique and more precise observation of the rigid and flexible regions occurring in proximity. Two more specific behaviours, mobility and deformability, can be hidden by the ‘term’ flexibility. A rigid region trapped between two flexible regions is collectively called a ‘mobile region’. In contrast, a deformable region can be defined by the inclusion of a flexible region trapped between two rigid regions [36]. These additional classifications allow for a more precise definition of local protein conformation and even add a continuum between the rigidity and flexibility of ordered proteins and disordered regions [37,38]. These properties are important, especially in the context of V_H_Hs docking, where in most algorithms, only the CDR loops are considered flexible regions for binding.

The current large-scale study of these domains aims to provide a better understanding of different properties such as flexibility, mobility and deformability in different regions to identify unexpected underlying differences between V_H_H domain trajectories.

## 2. Results

### 2.1. Dataset Description

**Figure 1:**
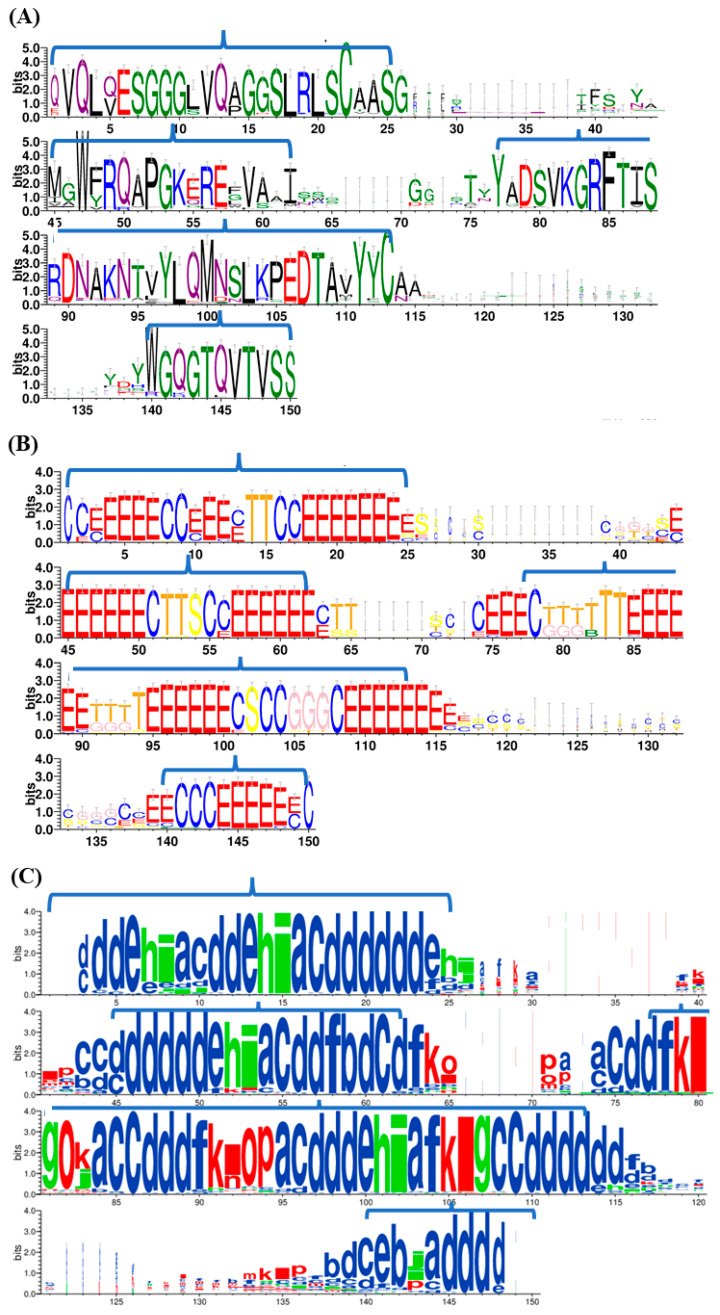
Sequence and structure characteristics in V_H_H dataset. Conservation of (**A**) amino acid residues, (**B**) secondary structures and (**C**) Protein Blocks. The four Framework regions are delineated in each figure.

88 V_H_H domains were chosen for classical MD simulations as in [20]. The 88 V_H_H sequences were aligned using ClustalOmega [39]. Figure 1A presents the sequence conservation in the aligned regions of the dataset. The extent of conservation is striking in the FRs and CDRs, as previously seen [20,40]. Additionally, the conserved residues such as C22, W45, F85 and C113 are observed (see the multiple sequence alignment in Figure S1).

Figure 1B provides the corresponding secondary structures at each aligned position. The β- strands forming the β-sheets of immunoglobulin fold as seen in alignment positions 4–9, 10–13, 17–24/25, 45–50, 56–62, 75–78, 85–90, 95–100, 109–115, 139–149, respectively. The connecting loops within FRs are also well conserved in terms of secondary structures. These loops are often associated with conserved turns and bends (FR1, positions 14–15, FR2, positions 52–54, FR3, from positions 79 to 84 and 91 to 94). Even with 3_10_-helix (FR3, positions 105–107), the FRs are well conserved and can be identified clearly. As expected, the CDR1 region (positions 26–44), CDR2 region (positions 63–74) and CDR3 region (positions 113–140) show secondary structure conservation, mainly in the positions close to FRs that showed some slight conservation with the extension of β-sheets (positions 44, 63, 75–77, 114–116, and 139). A turn is observed at the N-Cap region of CDR2 (positions 64–65). Interestingly, one can note the specific region (see alignment at positions 91–95) that is debated in the literature to be an additional fourth complementarity-determining region named CDR4 [41]. This region shows a conserved amino acid sequence (Figure 1A) but a slightly less conserved SSE signature (in Figure 1B). It is reported as a potential fourth CDR [42]. Position 83 (in Figure 1B) in 20% of the structures shows a β-bridge, which could be explored further.

Another way to look at local protein conformations is to use a structural alphabet such as Protein Blocks (PBs, [43]). It describes more precisely the local conformation [44]. The PB alignment shown in Figure 1C represents the corresponding PB assignment at each residue position of a given V_H_H domain aligned according to the MSA, where 10–20% diversity is observed in FR1 (positions 3–25), FR2 (45–62) and FR3 (77–113) regions, especially in the loop between the β-stands (represented by the Protein Block *d*). The PB analysis shows no residue is associated with the coil state. As expected, the CDRs show no particular PB conservation, even if slightly higher conservations in the PB series are seen in positions 71–72.

### 2.2. Assessment of Flexibility

Both experimental and simulated flexibility were analysed to gain insights into the true nature of conformational flexibility at each position of the V_H_H dataset. The (experimental) flexibility in these domains was assessed by looking at the distribution of normalised Cα B-factors (see Figure S2A). It presents an expected extreme distribution with a classical median value of −0.20. Low (negative normalised) B-factor values are considered rigid, while higher positive values are considered flexible.

The simulated flexibility came from four independent replicates for each V_H_H, leading to eighty-eight simulations of 1μs concatenated trajectories. It is classically analysed using Root Mean Square fluctuations. Comparison of normalised B-factors and normalised RMSF (see Figure S2B) shows a correlation coefficient of only 0.42 (equivalent to previous analyses [45,46]). Another measure of conformational diversity based on Protein Blocks, namely *N*_eq_, was also used to quantify position-wise conformational diversity. FRs have a median value of 0.63, 0.51, 0.54 and 0.51 Å (see Figure S3A–D). For CDRs, it is slightly higher, with 0.96, 0.70 and 0.78 Å for CDR1, CDR2 and CDR3, respectively (see Figure S3E–G). These results agree with the general idea that CDRs are more flexible than FRs. Nonetheless, this result must be interpreted cautiously as some FRs have high RMSF values, while CDRs can be associated with low RMSF values.

The median values for *N*_eq_ in FR1, FR2, FR3 and FR4 are all 1.0 (see Figure S4A–D). The most rigid FR is FR2 with no change (*N*_eq_ is always 1); while, for all others, some *N*_eq_ values can be higher than 2 and sometimes 3, with certain underlying plasticity. Rigidity order can be proposed with FR2 > FR4 > FR3 > FR1.

The median of *N*_eq_ values for CDR1, CDR2 and CDR3 are 1.35, 1.12 and 1.18, respectively (see Figure S4E–G); almost 50% of residues in the CDRs do not show any conformational diversity during the simulation. This shows the importance of considering metrics other than Cα RMSF that provide a simplified version of the flexibility, and that some mobile/deformable (frustrated) regions can be found in CDRs.

Comparison of Cα RMSF and normalised Cα B-factors show weak correlation for FRs, 0.50, 0.68, 0.51 and 0.60 for the FR1, FR2, FR3 and FR4 regions, respectively (see Figure S5A–D). This correlation decreases again with values of 0.42, 0.32 and 0.29 for CDR1, CDR2 and CDR3 regions (see Figure S5E–G), highlighting the fact that most complex links are to be found in CDRs.

### 2.3. Flexibility at Each Residue Position

**Figure 2:**
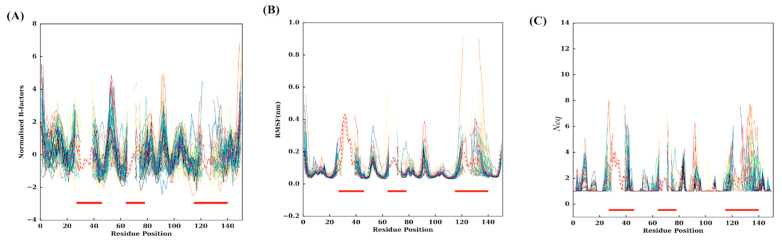
Flexibility metrics at each residue position. (**A**) Representation of normalised Cα B-factors; the *x*-axis is the residue position in the MSA and the *y*-axis the normalised Cα B-factor values. (**B**) Representation of Cα RMSF values. (**C**) Representation of *N*_eq_ values. The three CDR regions are highlighted using three red-coloured regions at the bottom of the plots. The average values of each metric are shown in dotted red lines.

**Figure 3:**
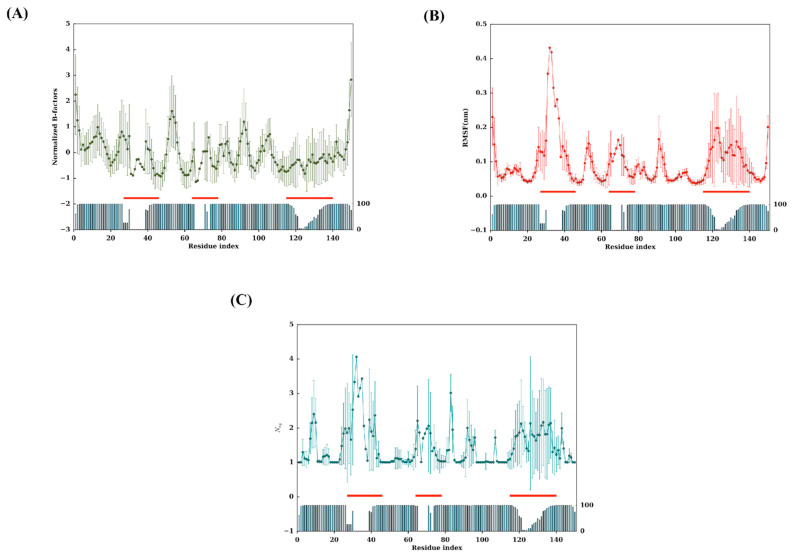
Tendencies of flexibility metrics at each residue position. Mean and standard deviation of (**A**) normalised Cα B-factors, (**B**) Cα RMSF and (**C**) *N*_eq_. Occurrence is shown as a histogram. CDR positions are shown as ref lines.

**Figure 4:**
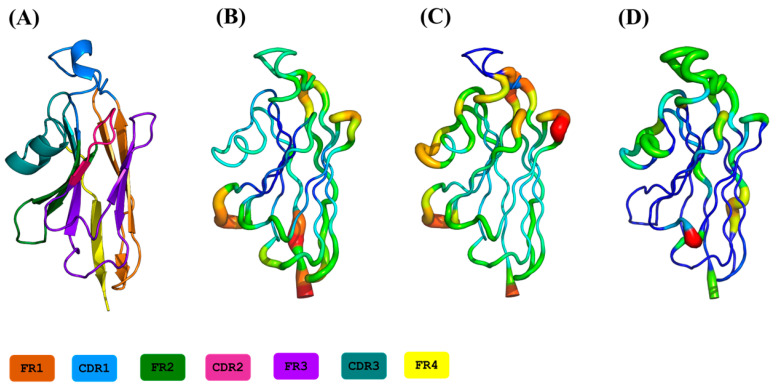
Representation of mean values of flexibility metrics onto a 3D structure of a V_H_H. (**A**) coloured FRs and CDRs, (**B**) mean normalised Cα B-factor values, (**C**) mean Cα RMSF values and (**D**) mean *N*_eq_ values of all V_H_Hs.

The experimental flexibility of each residue was analysed with normalised B-factors. The values were computed according to the corresponding positions of the MSA, i.e., a residue must be present to be taken into account in the average value. Figure 2A shows all individual 88 V_H_Hs, while Figure 3A presents extracted information with mean values and associated standard deviation. Interestingly, higher normalised Cα B-factor values are found mainly in FRs (see Figure 4A for a 3D depiction of FRs and CDRs). The highest value is FR2, followed by FR3 and FR1. Some regions of FRs are associated with low values (i.e., position 20 for FR1, position 45 for FR2 and 101 for FR3). CDRs rarely have flexible values. This result is really counterintuitive, but it should be noted that when V_H_Hs are in complex, the CDRs are highly rigid; the FRs are then more flexible than the latter as they are not blocked in interactions.

Analysis of RMSF (see Figure 2B, Figure 3B and Figure S3B) shows high values in RMSF in the CDRs (23–44, 63–74 and 113–139 MSA positions). Interestingly, the FR2 region (46–52) and FR3 (77–79 and 89–95) are also associated with high values. At first glance, the whole of FR2 (18 residues) is highly flexible except for two regions between 44–47 and 55–63. The region 89–95 is a small loop in the FR3, the additional fourth complementarity-determining region (CDR4) (also shown as the purple loop in Figure 4A and the thicker putty red region in Figure 4C [41]; it does not show amino acid sequence variation and SSE variation, but it is highly flexible in terms of RMSF.

We further assessed the 88 V_H_H MD trajectories by assigning Protein Blocks to each snapshot of each concatenated trajectory for changes in PB frequencies at each position during simulations. The resultant PB entropy calculated from PB frequencies at each position for each V_H_H concatenated trajectory is shown in Figure 2C. Surprisingly, some of the CDR1 residues in some trajectories at the termini of the loop (loop beginning 23–25 and ending 38–40 residue positions) show high *N*_eq_ values, as seen in the CDR3 region. Other amino acid regions such as positions 5–10 and 15–18 in FR1, 55–60 in FR2, 90–100 and 103–105 in FR3 show higher values of *N*_eq_ (closer to two most often but may increase up to four), suggesting that these regions could be the flexible regions trapped between two rigid regions, and might play a role in overall motion. The mean *N*_eq_ values are shown in Figure 3C to understand the trend in V_H_H trajectories. Similar to the putty representations for mean B-factors and mean RMSF values, the putty representation for *N*_eq_ values at each position is illustrated in Figure 4D. While the blue-coloured regions represent no change in the PB assignment, the green, yellow and red regions represent a higher mean *N*_eq_ value at respective positions.

### 2.4. Clustering of V_H_H Trajectories

**Figure 5:**
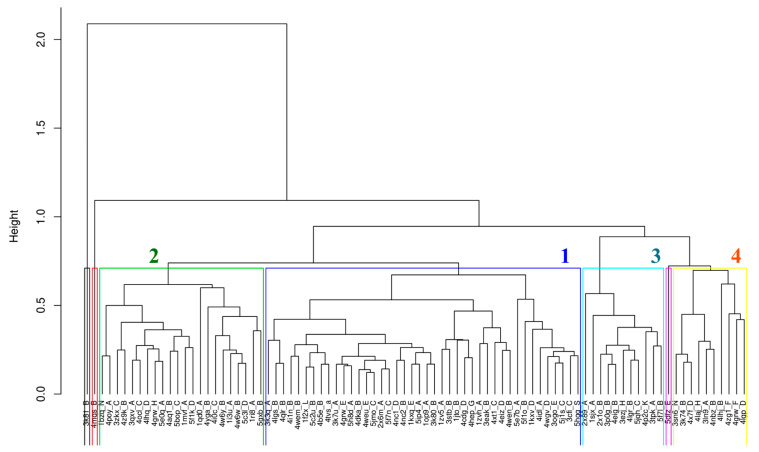
Hierarchical clustering of V_H_H trajectories using RMSF. The different RMSF clusters are demarcated using coloured boxes, and their cluster number is marked accordingly.

To investigate the underlying trends in dynamics, the 88 trajectories were clustered using hierarchical clustering using RMSF values, which resulted in four dense clusters and three singletons, as presented in Figure 5. The four dense clusters will be referred to henceforth as RMSF clusters. The largest RMSF cluster (blue color, 42 V_H_Hs, 47.7%) is associated with nearly half of V_H_H trajectories; it is close to RMSF cluster 2 (green color, 22 V_H_Hs, i.e., 25.0%). RMSF cluster 3 (cyan color, 11 V_H_Hs, 12.5%) and RMSF cluster 4 (yellow color, 10 V_H_Hs, 11.3%) are separated from the first two and related to one singleton (pink color), while the two singletons are clear outliers.

**Figure 6:**
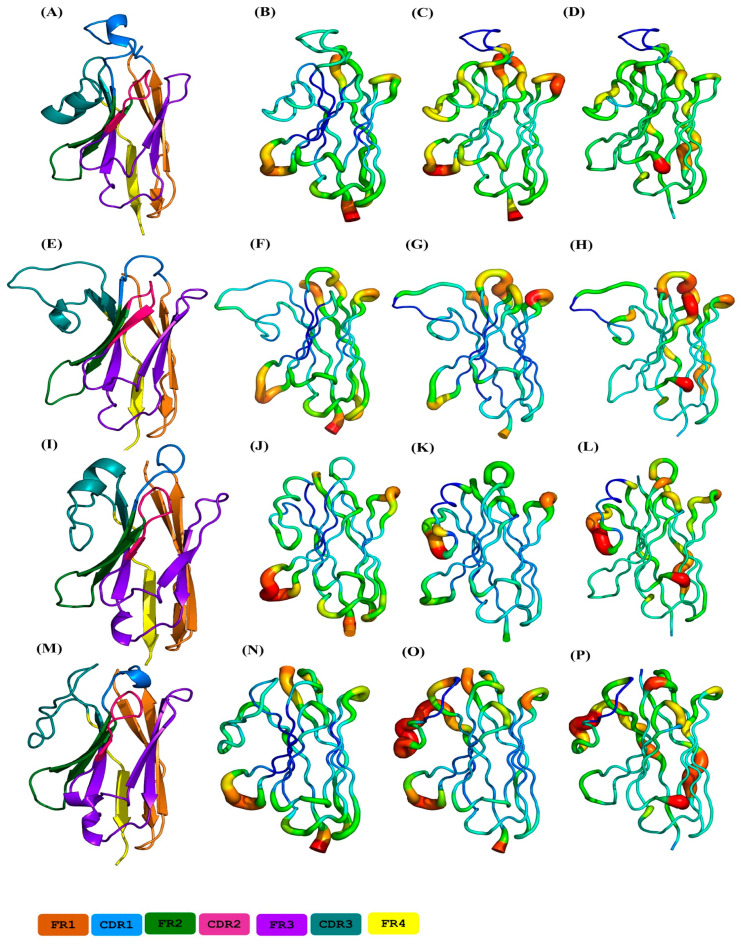
Representation of mean values of flexibility metrics onto a 3D structure of a V_H_H from each cluster. (**A**–**D**) RMSF cluster1, (**E**–**H**) RMSF cluster 2, (**I**–**L**) RMSF cluster 3, (**M**–**P**) and RMSF cluster 4, with (**A**,**E**,**I**,**M**) are coloured coded FRs and CDRs, (**B**,**F**,**J**,**N**) mean normalised Cα B-factors, (**C**,**G**,**K**,**O**) mean Cα RMSF values, and (**D**,**H**,**L**,**P**) mean *N*_eq_ values.

This classification is made on RMSF value along the V_H_H protein sequences. The average distribution of RMSF is shown in Figure 3B and is also visualised in Figure 5C on the 3D structures. The structure parts concerned with the RMSF values of each RMSF cluster are now presented in Figure 6C,G,K,O, respectively (see also Figures S6–S8).

The evaluation of the four RMSF clusters underlines the contributions of different regions to the clustering. CDR3 (MSA positions 113–139) shows the most significant variability between the four RMSF clusters. At these positions, the RMSF values for RMSF clusters 3 and 4 (see Figures S6C,D and S7C,D) are much higher than those for RMSF clusters 1 and 2 (see Figures S6A,B and S7A,B). RMSF cluster 1 is the closest to the general distribution, with only a slight increase in rigidity (on average, around 1.0 Å, see Figure 3B and Figure S8A), RMSF cluster 2 is the most rigid (decrease of RMSF value around 1.0 Å) while its extremities are more flexible. In contrast, RMSF cluster 3 is the most rigid for CDR3 (increase around 1.5 Å). There is, therefore, a clear gradient from flexible to rigid RMSF cluster 2 > cluster 1 > cluster 4 > cluster 3 for CDR3. Surprisingly, the long CDR3s, which are therefore the rarest, have shown specificities. For the positions 125–127 (of the MSA), the clusters behave contrary to the general trend. These positions have less than 10% occurrence in any of the clusters. RMSF clusters 3 and 4 are more flexible, while the other two are more rigid when C𝛼 RMSF and *N*_eq_ values are considered (see Figures S8–S11). It should be noted that this rigidity is relative, as CDR3 is a reasonably flexible area.

The second most contributing region is CDR1 (positions 25–44 of MSA). Most of the RMSF clusters are relatively close to the mean value of the distribution, and only RMSF cluster 2 has an increase in its rigidity (see Figure 6G and corresponding Figures S7B and S8B). RMSF cluster 1 has a slight increase in flexibility (around 0.5 Å); differences are negligible for the others (see Figure 6K,O, Figure S7C,D and Figure S8C,D).

The other regions show little specificity in their contribution to clustering. FR3 in its central part (position 90 in the MSA) offers a variation close to CDR1, with slight rigidification for RMSF clusters 2 and 3 and slightly more flexibility for the other two.

CDR2 had little variability (low standard deviation) and thus had minimal variation in the clusters. Only the terminal parts of RMSF clusters 1 and 2 have some variations (slightly more flexible for RMSF cluster 1, somewhat more rigid for RMSF cluster 2).

Interestingly, the analysis of associated normalised C𝛼 B-factor presented in Figure 6B,F,J,N, respectively (see also Figures S9–S11), reveals only a partial correlation with RMSF values. RMSF cluster 1 differs only slightly from general V_H_H tendencies (see Figure 3A) for CDR3 and CDR2, but the N-cap of CDR1 is largely more rigid (around 0.8 Å, see Figures S9A, S10A and S11A). RMSF cluster 2 shows more rigid residue in CDR3 (around 1.0 Å, see Figures S9B, S10B and S11B). For RMSF clusters 3 and 4, differences are more striking. RMSF cluster 3 has more rigid residues in FR1 and CDR1 N-cap (around 1.5 Å, see Figures S9C, S10C and S11C); CDR2 and CDR3 are more flexible; RMSF cluster 4 had a succession of more rigid, more flexible and finally more rigid residues in CDR1 (around 1.0 Å, see Figures S9D, S10D and S11D); CDR2 is slightly more rigid at its C-terminal positions, as is CDR3. It shows a somewhat different view than with RMSF data.

Localisation of protein parts concerned by the RMSF values of the different RMSF clusters are now presented in Figure 6D,H,L,P, respectively (see also Figures S12–S14). Figure 3C showed that CDRs have average *N*_eq_ values around two (MSA positions 25–43, 63–74 and 114–139), and some regions of FRs too (5–8, 55–60, 83–85, and 90–95). Figure 2C also showed that some individual V_H_H could reach relatively higher values, e.g., CDRs 1 and 3 can sometimes reach an impressive *N*_eq_ value of eight, i.e., a value mainly associated with disordered regions (see [37]) where large-scale fluctuations can be seen between V_H_H domains.

This also allows us to see what has been considered in the clustering. It underlines how FR1’s main specificity is an increase of flexibility for RMSF and B-factor of cluster 3 and a decrease for cluster 2. It also indicates that CDR3 is crucial once again, with an increase in rigidity for B-factor, RMSF and *N*_eq_ of cluster 3 and RMSF and *N*_eq_ of cluster 4, while the latter had an increase in flexibility in B-factor. The C-terminal loop in the FR2 region and the N-terminal loop in the FR3 region (or the presumed CDR4 loop) behave differently with respect to the metrics. This difference is the main inference drawn from this analysis: that the c-terminal loop included in the FR2 region shows high RMSF values, although the changes in backbone conformational flexibility assessed using *N*_eq_ remain hardly noticeable. This is the classic case of mobility where the whole loop fragment is observed to be in motion with no apparent change in its backbone conformations. The other example is the N-terminal loop enclosed inside the FR3 region. This loop shows higher RMSF and *N*_eq_ values but not at all the residue positions, making this region a true example of a deformable region (see also Table S1). An important point to underline is that B-factor, RMSF and *N*_eq_ do not have a direct link and are sometimes slightly opposed in terms of dynamical tendencies [36,47,48].

### 2.5. Backbone Conformational Changes in Terms of Protein Blocks

**Figure 7:**
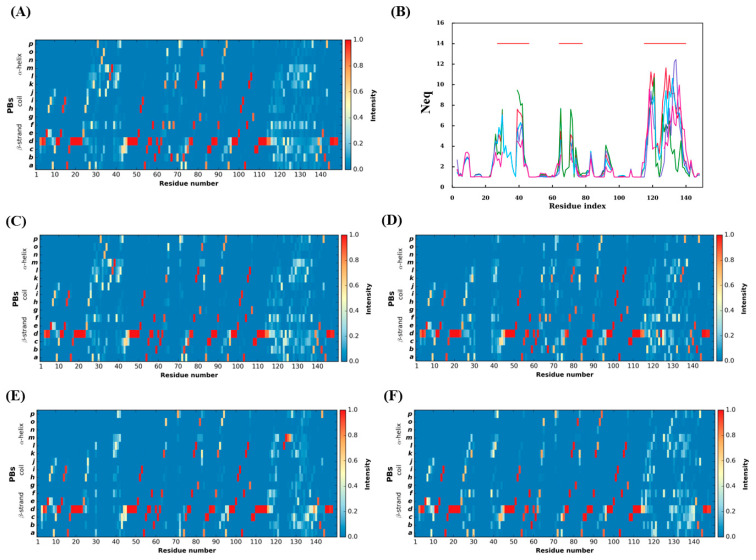
Local backbone diversity at the light of PBs. (**A**) PB map of all V_H_H trajectories aligned according to MSA, (**B**) *N*_eq_ values (red line—all V_H_H trajectories, sky blue—V_H_H trajectories belonging to RMSF cluster 1, green—V_H_H trajectories belonging to RMSF cluster 2, purple—V_H_H trajectories belonging to RMSF cluster 3 and pink—V_H_H trajectories belonging to RMSF cluster 4), PBs map of V_H_H trajectories belonging to (**C**) from RMSF cluster 1, (**D**) from RMSF cluster 2, (**E**) from RMSF cluster 3 and (**F**) from RMSF cluster 4. The *x*-axis represents the residue positions, and the *y*-axis represents the types of PBs or the *N*_eq_.

PBs occurrence observed for the 88 V_H_Hs trajectories is shown in Figure 7A. This figure reaffirms the general idea that the CDRs (MSA positions 25–47, 62–75, 113–140) are conformationally diverse, and FRs are less varied, as seen previously (5–10, 79–82, and 91–95). These last FR zones thus seem to have two different sets of conformations. The classical β-stand representative PB *d* is consecutively (although with interruptions) seen in the FR regions with a high occurrence, e.g., 72 positions with PB *d* represented more than 80% of the time. The regions 48–62 and 98–113 are the two regions which have retained the most conserved PBs. Figure 7C–F provide the PB occurrence associated with each RMSF cluster, while Figure 7B shows the *N*_eq_ of each RMSF cluster. This lets us see that conformational diversity is demarcated with high *N*_eq_ values, e.g., *N*_eq_ values between 8 and 12 in CDR1 and CDR3 regions at almost all the residue positions. Hence, FR1, FR2 and FR4 are highly similar in terms of *N*_eq_ for all clusters, while FR3 (around MSA position 95) is more complex with *N*_eq_ values less than two for RMSF cluster 4 (pink line), so slightly rigid, while the others can reach ‘four’ as in case of RMSF cluster 2 (in green), e.g., flexible. Around MSA position 85 of the same FR, they all have the same *N*_eq_ value of three as that of MSA position 10 of FR1. They are the most prominent positions in terms of *N*_eq_ for FRs. In FR2, RMSF cluster 2 has a higher value than the average distribution (in green 8, i.e., disorder vs. high flexibility in red 6, see Figure 7B), while RMSF cluster 4 is only at 3. It is observed that the low *N*_eq_ values of position 70 are mainly due to the low occurrence number at this position for RMSF cluster 2. For CDRs, the situation is, as expected, more complex.

**Figure 8:**
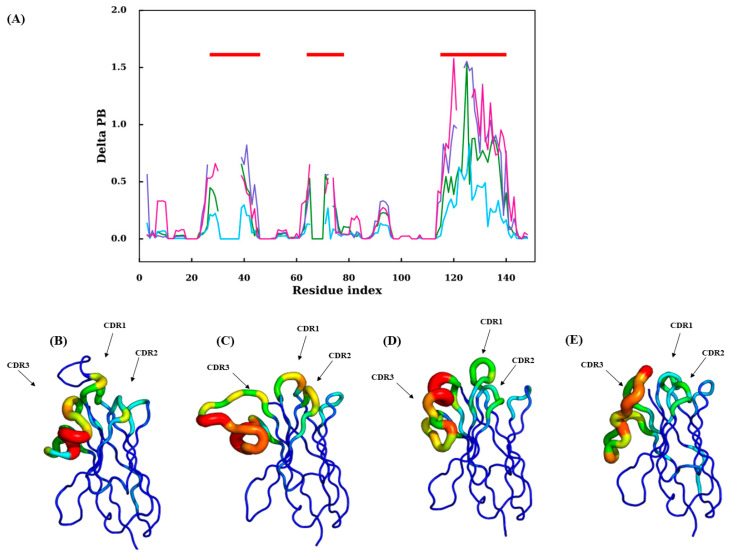
PBs’ differences in terms between each RMSF cluster. (**A**) ΔPB (with RMSF cluster 1 in sky blue, with RMSF cluster 2 in green, with RMSF cluster 3 in purple and with RMSF cluster 4 in pink); CDRs are shown with red line. 3D visualisation on structures of ΔPB values for (**B**) RMSF cluster 1, (**C**) RMSF cluster 2, (**D**) RMSF cluster 3, and (**E**) RMSF cluster 4.

Interestingly, the same antagonism can be observed in CDR1 for RMSF clusters 2 and 4 (e.g., in the C-terminal region, *N*_eq_ > 9 for RMSF cluster 2, *N*_eq_ of 7 on average and of 4 for RMSF cluster 4). For CDR3, the situation is different, with high average *N*_eq_ corresponding to very different disorder conformations. Here, it is mainly RMSF cluster 4 that is more rigid, with *N*_eq_ values around six or less. Very few qualitatively distinct regions are seen when these four PB maps are compared. As a consequence, a comparison of amino acid frequencies and PB frequencies in the structural dataset and PB frequencies in the cumulative PB frequencies from trajectories belonging to each cluster were assessed in order to obtain clarity (the sequence logos for amino acid and Protein Blocks for the four clusters are shown in Figures S16 and S17). To understand how each cluster differs from the whole dataset, the difference in the frequencies of amino acid or PB at each residue position of that cluster is subtracted from that of the whole dataset. The sum of absolute values of the resultant frequency difference at each residue position gives a quantitative estimate of the variability in amino acids or PBs (ΔAA and ΔPB overlays of all four clusters are seen in Figure S18A,B, respectively). At first glance, one can appreciate the stark differences in amino acid and PB frequencies, suggesting a certain tolerance level for backbone conformations to amino acid variations in the FR regions (1–25, 45–63, 77–113, 140–150) albeit with some deviations in the C-terminus loops inside the FR 1, 2 and 3 and the CDR4 (N-terminal loop) present in FR3 region. This difference is also reflected in the PB analyses of trajectories shown in Figure 8. A correlation between amino acid frequency difference and PB difference of the four clusters in most residue positions confirms that FRs show less diversity in both AA and PBs than CDRs (see Figure S20A–D).

### 2.6. Regions-Wise Correlation between Amino Acid Sequence and PB Sequences

To understand which residue positions, in different regions, show the most variability in terms of amino acids (AA) and PBs, and how they are correlated, a region-wise examination of different residue positions is presented below.

#### 2.6.1. Framework Region 1

In FR1, seven residue positions for ΔAA and five residue positions for ΔPB show no change with respect to the dataset. Gly8, Gly9, Gly15, Ser17, Leu18, Leu 20 and the characteristic Cys 22 are the conserved positions in all the clusters. Regarding PBs, residue positions 19, 20, 21, 22 and 23 are clearly observed to be the extended confirmation of PB ‘d’ in all the clusters. The residue positions between 10 to 17 also show very few variations in terms of PBs, as the ΔPB in this region for all the clusters is in the range of 0.02 to 0.04, suggesting that the PBs between a particular cluster and the dataset are not significantly diverse. This observation is corroborated by the dynamics of VHH belonging to each cluster in this region (1–25) in Figure 8A. This analysis reveals, through two types of observations, that Gly8 and Gly9 positions should not be altered because they preserve the flexibility in the region as denoted by the diverse PB set (PB a, e, i and h). Whereas, positions 19 and 21 tolerate mainly AA substitutions that are hydrophilic (Ser, Thr, Arg, Lys).

#### 2.6.2. Framework Region 2

It comprises the region between 45–62 residue positions in the MSA. There is only one conserved amino acid residue position: the Trp47. Residue position 52 is almost always represented by Proline except in limited cases where Threonine is found in V_H_H belonging to cluster 3. The residue positions 48, 55, 56 and 58 are those known to undergo hydrophilic amino acid substitutions compared to their VH counterparts. Regarding PBs, positions 46–50 and 57 show no variations between clusters and the dataset. Surprisingly, none of the residue positions show more than 0.3 values in ΔPB. This is also observed in PB assignments of trajectories in the regions 45–62 in Figure 7A, where we see mostly conserved PB assignments indicated by the intensity of the red colour.

#### 2.6.3. Framework Region 3

The region comprises 77–113 residue positions in the MSA. It has a few conserved residue positions [49] such as the Cys113, Arg85, Phe86, Ser89, Asp91, Leu99, Leu104, Asp109, Thr110 and Ala111. The change in ΔAA never exceeds 0.45, and the ΔPB is notably high only for one residue position 93, which is in the hypothesised CDR4 or the DE loop. Next, almost all the residue positions from 98 to 113 show very insignificant change in ΔPB values, suggesting this region is conserved in terms of local conformation (see Figure S20C). Another notable observation is that this is the region that does not undergo high AA substitutions (>0.4 for more than 90%) or changes in local conformations (>0.2 for more than 95%), suggesting it is the most conserved region in the structure. This is also reflected in the dynamics in Figure 8A; in the FR3 region between 77–113, cluster 4, cluster 3 and cluster 1 show higher ΔPB values in the CDR4 region (89–93) and at the beginning of the FR3 region.

#### 2.6.4. Framework Region 4

It comprises a region between 140–150. It is eleven residues long and is the most conserved region, with seven residue positions, 141, 143, 144, 146, 147, 149 and 150, showing the same frequencies in all the clusters compared to the dataset. Residue 140 is mostly tryptophan, other than arginine, lysine and tyrosine occurring in a few domains. The most varying position of the alignment is 140 in cluster 3 and cluster 4. Regarding ΔPB, the residue positions 146 and 147 are almost always conserved. This is also reflected in PBs in the dynamics (see Figure 7B); PB diversity is hardly seen in clusters 1, 2, and 3 (*N*_eq_ ~ 3). Only cluster 4 shows higher values of *N*_eq_.

#### 2.6.5. Complementary Determining Regions

For CDRs, it is not as simple as for FRs to perform the analysis as mentioned above. As expected, the ΔAA and ΔPB are uncorrelated. However, if cluster-wise values are considered (see Figure S20E–G), cluster 3 and cluster 4 show higher values in all three CDRs, which are generally observed in the literature too.

## 3. Discussion

This study carried out a large-scale conformational exploration of V_H_H domains for the first time. A set of 88 V_H_H domain trajectories of 1μs were analysed with the principal aim towards understanding the flexibility in different regions of V_H_H domains, using classical methods such as Cα RMSF and innovative in-house methods such as Protein Blocks.

Substantial analyses were performed to reveal the differences in metrics used to denote flexibility experimentally and theoretically. A startling observation is shown in Figure 4, where there is a discrepancy between the mean B-factor (Figure 4B), mean RMSF (Figure 4C) and the mean *N*_eq_ values (Figure 4D). This Figure underlined that regions with high B-factor values can be associated with low RMSF and *N*_eq_ values. It suggests that not all residues with high-normalised B-factor values must be always considered conformationally diverse residues. In previous studies on a large set of globular proteins, it was determined that B-factor and RMSF have a correlation of 0.42–0.45, while it is less than 0.15 with *N*_eq_ [50,51,52]. These values may seem low, but the experimental approaches and simulations each have their own limitations (crystalline contact, locally limited sampling, etc.). The differences between RMSF/B-factor and *N*_eq_ are expected; the first being values calculated globally, while the *N*_eq_ is local (over five residues) [36,46].

Hierarchical clustering was then used to cluster the V_H_H trajectories using RMSF values. The distance calculation was guided by the MSA of V_H_H sequences in the dataset; only RMSF values of aligned residue positions were used. Four dense clusters show varying degrees of flexibility in FRS and CDRs. A first look at the normalised B-factors of structures from these four clusters reveals almost entirely flexible regions in FR4 and FR1, except for residue regions (21–25). The C-terminal loop in FR2 is almost always flexible in all four clusters (54–56), see Figure 5. Even the so-called CDR4 loop is flexible in all four clusters. When the RMSF values were compared in these clusters in the flexible regions mentioned above, they were almost always in the regions mentioned above except for a few residues at the extremities. This often gives a wrong impression about conformational diversity.

To enable a much deeper understanding of conformational diversity, the residues in the three CDR loops are categorised based on two thresholds for normalised RMSF (2.0) and *N*_eq_ (3.0) to classify the mobile and flexible regions. The quadrant with normalised RMSF > 2.0 and *N*_eq_ *>* 3.0 was considered flexible. The quadrant with normalised-RMSF > 2.0 and *N*_eq_ < 3.0 was considered mobile. Both regions in all the CDRs showed normalised B-factor values in the positive and negative range, suggesting that B-factors alone as a criterion for flexibility is insufficient.

We attempt to further delineate flexibility in CDRs by assessing the correlation between normalised RMSF and *N*_eq_ variables, as shown in Figure S18A,D,G for CDR1, CDR2 and CDR3, respectively. Four regions in these plots were delineated using two cut-off values arbitrarily determined. This value was two for normalised RMSF and three for *N*_eq_. This choice is based on our previous experiences of analysis of classical MD simulations of ordered and disordered regions of proteins. We have focused on two quadrants with high-normalised RMSF (above two): (i) the one with low *N*_eq_ below three, the residues are considered mobile, and (ii) the one with *N*_eq_ above three, where the residues are considered flexible. The question is whether these behaviours are already seen in experimental values, namely B-factors. Hence, the normalised B-factors for these residues from the original crystal structures are shown in the distributions next to the scatter plots. The second vertical panel is for the flexible region with CDR1 (see Figure S21B), CDR2 (see Figure S21E) and CDR3 (see Figure S21H). The third vertical panel (Figure S21C,F,I) is for the mobile region in the same order.

Both panels for all the CDRs show a range of normalised B-factors from negative to positive, suggesting that regions, which are otherwise classified as rigid based on normalised B-factors, show conformational diversity. Median values are also equivalent, so that no difference can be observed directly from these experiments.

After further examination of the differences in amino acid content in the two quadrants, surprisingly, both regions had the most similar presence of amino acids; however, some were exclusive to each group. For example, glutamate, methionine, proline and histidine were exclusive to the flexible quadrant, whereas asparagine, isoleucine and tryptophan were exclusive to the mobile quadrant for CDR1. Interestingly, proline is present in the mobile region for CDR2 along with aspartate, and like in the case of CDR1, glutamate is exclusive to the flexible region.

The amino acids cystine and lysine are exclusive to the flexible region of CDR3, and phenylalanine, asparagine, and methionine are exclusive to the mobile quadrant.

Our previous study observed that the distance between CDR1 terminal residues in the V_H_H domains is not conserved and can vary ±3 Å in the dataset. In this current study, we wanted to verify where the deviation is conserved in simulation in the case of CDR1 termini, and if there are any observable changes in CDR2 and CDR3.

**Figure 9:**
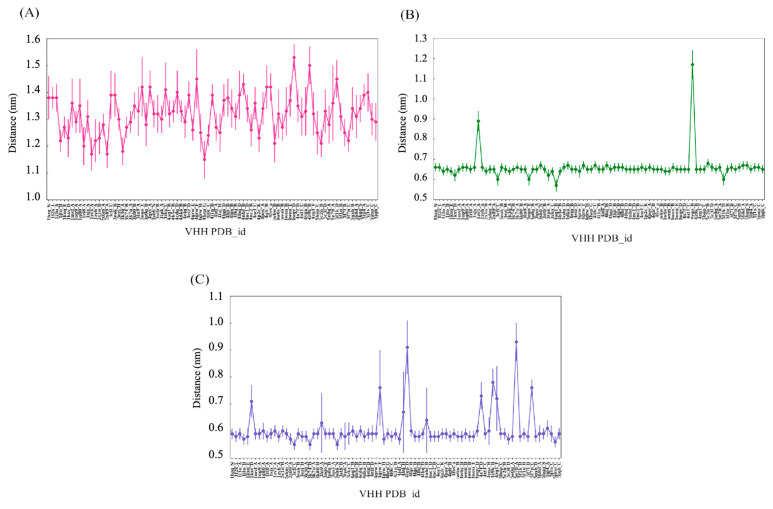
Mean of Distance between CDRs termini in each V_H_H trajectory. (**A**) CDR1, (**B**) CDR2 and (**C**) CDR3 termini.

This analysis is shown in Figure 8, where the mean and standard deviation in the termini length observed for all the CDRs for concatenated 1 μs trajectories of 88 V_H_H have been shown. The CDR1 termini length distributions shown in Figure 9A convey that there is, on average, 0.1 Å std for all the CDR1 in the dataset. However, it is strange to note that the mean extremity lengths in some cases are noticeably lower. In the case of CDR2 (Figure 9B), two V_H_H trajectories show a high mean termini distance compared to the rest of the trajectories. In the case of CDR3 (Figure 9C), cases show more than 0.1 Å differences in mean CDR3 termini distance. This analysis reveals that the CDR2 and CDR3 termini are less displaced due to each other’s terminal residue, whereas the CDR1 terminal residue shows much more preference towards the displacements.

The current study is the first to attempt to underline the RMSF variations used to classify entire domains as they are known to be influenced by amino acids at their respective positions, which in turn influence local conformational flexibility.

In our further analysis to specify the residue positions, which change local conformational flexibility in FRs, we looked at the correlation between amino acid frequency conservation and PB frequency conservation in a specific cluster with respect to the dataset. This analysis showed a fascinating observation (reference Figure S20A–D) that shows that the FR1 and FR4 show greater changes in ΔPB to ΔAA in the regions. In contrast, the FR2 region shows a lesser degree of PB (local conformational flexibility) diversity with higher diversity of AA in all clusters, most conspicuously in cluster 4. The most conformational conserved region is FR3; it does not show many variations in amino acids, except in the ‘CDR4 region’ (89–90 residue position in the MSA), which is also preserved in terms of PB assignments in structure and dynamics (Figure 8A).

**Figure 10:**
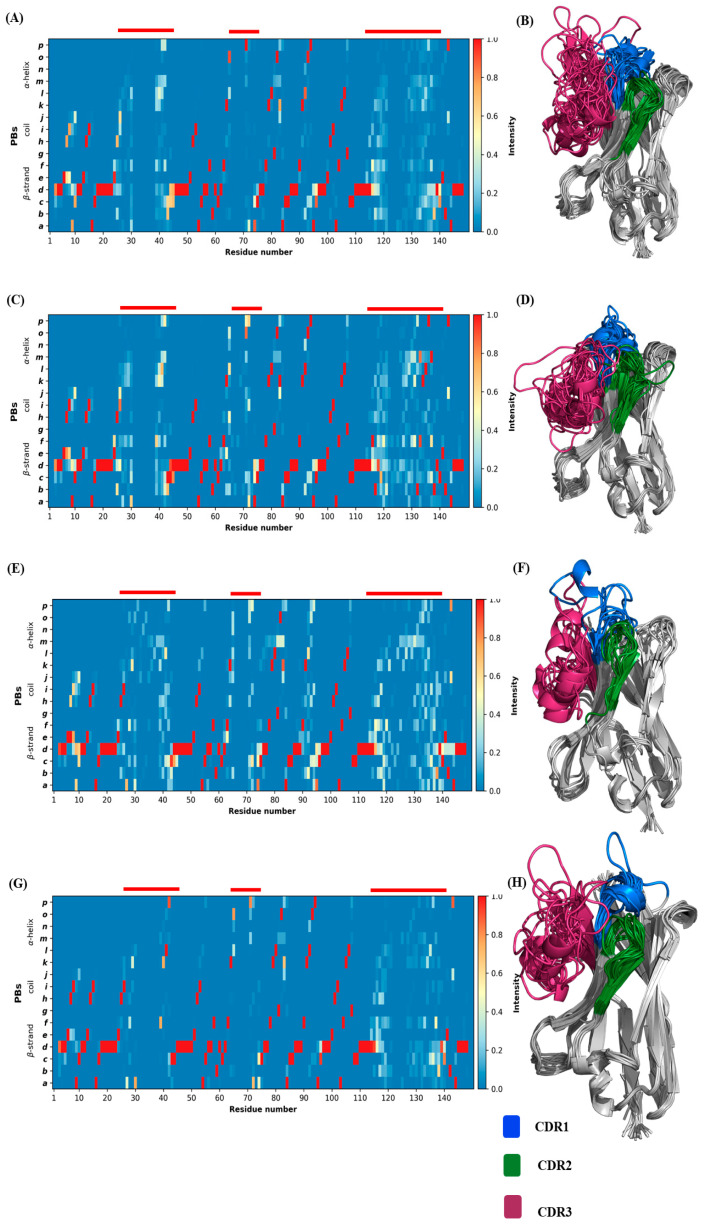
Representation of backbone conformations from trajectories clustered using average ΔPB. (**A**,**C**,**E**,**G**) are PB maps of the four ΔPB clusters, (**B**,**D**,**F**,**H**) are the visualisation of the structures.

Of course, our approach has shortcomings and could be improved by adding new structures of V_H_Hs, but also by continuing the analysis towards docking. An interesting point would be to test metrics other than a Euclidean distance. To understand the diversity in RMSF across the length of V_H_H, it is reflected in terms of PBs. We conducted another clustering analysis, this time using average ΔPB (see Figure S22). This analysis also resulted in four distinct clusters. A PB map of the concatenated trajectories and their initial starting structures are shown in Figure 10. A confusion matrix was calculated between the clusters obtained by RMSF and average ΔPB (see Table S2). This analysis revealed that the largest cluster in hierarchical clustering using RMSF values was distributed among all the four clusters obtained using average ΔPB as the criteria. The 42 cluster members from cluster 1 (RMSF clustering) are found to belong to cluster 1 (Average ΔPB clustering): 15, cluster 2 (Average ΔPB clustering): 11, cluster 3 (Average ΔPB clustering): 12 and cluster 4 (Average ΔPB clustering): 4. This brings us to ask whether local conformation-based clustering may provide more meaningful full comparisons between the dynamics of homologous domains, especially in V_H_H, to understand their behaviours.

## 4. Materials and Methods

### 4.1. Protein Structure Databank

We selected 88 V_H_H protein structures from the Protein Data Bank [17]. They are non-redundant, as described in [20]. Multiple Sequence Alignments were performed with ClustalOmega tool [39].

### 4.2. Molecular Dynamics

The MD protocol follows the same principle as our previous works [46,47]. The domains were simulated using GROMACS 2016.4 software [53,54] with the AMBERff99SBildn force field [55], with TIP3P water molecules added to solvate, surrounding the V_H_H in the centre of a dodecahedron periodic box with a side of at least 10 Å between the edge of the box and the protein. Hydrogen atoms were added to represent protonation states at pH 7. In each case, the system was neutralised, and then 150 mM of NaCl was added to match the physiological conditions. Again, the entire system’s periodic electrostatics was calculated using Particle Mesh Ewald (PME). The minimisation was performed using the steepest descent algorithm for 50,000 steps. An equilibration run was performed using an NVT ensemble to heat to 300 K using a modified Berendsen thermostat; then, an NPT was run with Parrinello−Rahman coupling for pressure control at 1 atm. All bonds were constrained with the parallel LINCS method, with short-range no bonded electrostatic interactions calculated with a cut-off of 10 Å and van der Waals with a cut-off of 10 Å. Each production run was completed for 250 ns total using a 2 fs time step in four replicates amounting to 1 μs for each protein. The coordinates and the velocities were calculated at every 10 ps interval.

### 4.3. Molecular Dynamics Analysis

The analysis of MD trajectories is performed using classic tools, such as the Root-Mean-Square Fluctuation (RMSF) of the Cα atoms using scripts from GROMACS software, and other more innovative approaches such as PBxplore [56], available on GitHub (https://pypi.org/project/pbxplore/ accessed on 8 January 2023). PBxplore allows it to assign Protein Blocks (see below) throughout the MD trajectories (see Method S1 for more details).

Protein Blocks (PBs) are a structural alphabet composed of 16 local prototypes [43]. PBs give a reasonable approximation of all local protein 3D structures [44]. PBs are very efficient in tasks such as protein superimpositions [57,58,59] and MD analyses [36], even for disorder proteins [38]. PB assignments are performed for each residue of the C-domain and over every snapshot extracted from MD simulations. The equivalent number of PBs (*N*_eq_) is a statistical measurement similar to entropy, representing the average number of PBs for a residue at a given position. *N*_eq_ is calculated as follows [43]:

$$N_{\mathrm{eq}}=\exp\left(-\overset{16}{\underset{x=1}{\sum }}f_{x}\ln f_{x}\right)$$

where *f_x_* is the probability of PB *x*. A *N*_eq_ value of 1 indicates that only one type of PB is observed, while a value of 16 is equivalent to a random distribution. To underline the main differences between any two sets of trajectories/structures for each position, the Δ*N*_eq_ value is computed. Δ*N*_eq_ is the absolute difference between corresponding *N*_eq_ values.

However, the same Δ*N*_eq_ value can be obtained with different types of PB in similar proportions. To detect a change in the PB profile, a Δ*PB* value was calculated. It corresponds to the absolute sum of the differences for each PB between the probabilities of a PB *x* present in the first and the second forms (*x* goes from PB *a* to PB *p*). Δ*PB* is calculated as follows [47]:

$$\Delta PB=\overset{16}{\underset{x=1}{\sum }}\left|\left(f_{x}^{1}-f_{x}^{2}\right)\right|$$

where *f*^1st^*_x_* and *f*^2nd^*_x_* are the percentages of occurrence of a PB *x* in respectively the first and the second system. A value of 0 indicates perfect PBs identity between the 1st and 2nd systems, while a score of 2 indicates a maximum total difference.

### 4.4. Protein Structure and Trajectory Visualisation

Visualisation of original structures was performed using PyMOL (The PyMOL Molecular Graphics System, Version 1.7.2 Schrödinger, LLC) [60,61]. The trajectories were visualised using VMD [62]. Secondary structure assignment was performed using DSSP (version 2.2.1 available at GitHub, https://github.com/cmbic/xssp accessed on 8 January 2023) with default parameters [63].

### 4.5. Metric Normalisation

Normalised B-factors were calculated from experimental B-factors as mentioned in the study [64], wherein the B-factor of the Cα atom was extracted for all the residues of the protein, and B-factor of (*i*th residue) was treated with the mean and standard deviation (*B_σ_* of all the B-factors of Cα atoms of a given domain like in the formula, a similar method was applied to normalised RMSF also):

$$B_{Norm}=(B_{i}-B_{\mu })/B_{\sigma }$$

### 4.6. Hierarchical Clustering of V_H_H Dynamics

RMSF were used to compare V_H_H dynamics and propose a clustering. A simple normalised Euclidean distance metric was used in our previous study [65,66]. Only positions with aligned amino acids are used for the distance calculation using the following formula:

$$d\left(v,w\right)=\sqrt{\frac{1}{n-m}\overset{n}{\underset{i=1}{\sum }}\left(v\left(i\right)-w\left(i\right)\right)}$$

*d*(*v*,*w*) is the distance between RMSF of V_H_H *v* and V_H_H *w*, *n* represents the total length of the alignment and *m* the number of gaps. From the distance matrix of *d* values for the 88 V_H_Hs, hierarchical clustering with a complete metric is performed with R software.

### 4.7. Scripting

All the scripts for analysing V_H_H structures were performed using Python 3.6 with NumPy library [67] and R 3.3.3 [68]. Sequence alignments were performed by the ClustalOmega tool (version 1.2.4) with default parameters [39] and visualised with Jalview (version 2.11.2.3) [69].

## Data Availability

Molecular dynamics trajectories are available on request.

## Supplementary Materials

The following supporting information can be downloaded at https://www.mdpi.com/article/10.3390/ijms24054511/s1.

## References

[1] Scully M., Cataland S.R., Peyvandi F., Coppo P., Knöbl P., Kremer Hovinga J.A., Metjian A., de la Rubia J., Pavenski K., Callewaert F. (2019). Caplacizumab treatment for acquired thrombotic thrombocytopenic purpura. N. Engl. J. Med..

[2] Jovčevska I., Muyldermans S. (2020). The therapeutic potential of nanobodies. BioDrugs Clin. Immunother. Biopharm. Gene Ther..

[3] Senolt L. (2019). Emerging therapies in rheumatoid arthritis: Focus on monoclonal antibodies. F1000Research.

[4] Huo J., Le Bas A., Ruza R.R., Duyvesteyn H.M.E., Mikolajek H., Malinauskas T., Tan T.K., Rijal P., Dumoux M., Ward P.N. (2020). Neutralizing nanobodies bind SARS-CoV-2 spike rbd and block interaction with ace2. Nat. Struct. Mol. Biol..

[5] Wrapp D., De Vlieger D., Corbett K.S., Torres G.M., Wang N., Van Breedam W., Roose K., van Schie L., Hoffmann M., Pöhlmann S. (2020). Structural basis for potent neutralization of betacoronaviruses by single-domain camelid antibodies. Cell.

[6] Chen F., Liu Z., Jiang F. (2021). Prospects of neutralizing nanobodies against SARS-CoV-2. Front. Immunol..

[7] Güttler T., Aksu M., Dickmanns A., Stegmann K.M., Gregor K., Rees R., Taxer W., Rymarenko O., Schünemann J., Dienemann C. (2021). Neutralization of SARS-CoV-2 by highly potent, hyperthermostable, and mutation-tolerant nanobodies. EMBO J..

[8] Hanke L., Vidakovics Perez L., Sheward D.J., Das H., Schulte T., Moliner-Morro A., Corcoran M., Achour A., Karlsson Hedestam G.B., Hällberg B.M. (2020). An alpaca nanobody neutralizes SARS-CoV-2 by blocking receptor interaction. Nat. Commun..

[9] Koenig P.A., Das H., Liu H., Kümmerer B.M., Gohr F.N., Jenster L.M., Schiffelers L.D.J., Tesfamariam Y.M., Uchima M., Wuerth J.D. (2021). Structure-guided multivalent nanobodies block SARS-CoV-2 infection and suppress mutational escape. Science.

[10] Schoof M., Faust B., Saunders R.A., Sangwan S., Rezelj V., Hoppe N., Boone M., Billesbølle C.B., Puchades C., Azumaya C.M. (2020). An ultrapotent synthetic nanobody neutralizes SARS-CoV-2 by stabilizing inactive spike. Science.

[11] Wu Y., Li C., Xia S., Tian X., Kong Y., Wang Z., Gu C., Zhang R., Tu C., Xie Y. (2020). Identification of human single-domain antibodies against SARS-CoV-2. Cell Host Microbe.

[12] Xiang Y., Nambulli S., Xiao Z., Liu H., Sang Z., Duprex W.P., Schneidman-Duhovny D., Zhang C., Shi Y. (2020). Versatile and multivalent nanobodies efficiently neutralize SARS-CoV-2. Science.

[13] Xu J., Xu K., Jung S., Conte A., Lieberman J., Muecksch F., Lorenzi J.C.C., Park S., Schmidt F., Wang Z. (2021). Nanobodies from camelid mice and llamas neutralize SARS-CoV-2 variants. Nature.

[14] Verkhivker G.M., Agajanian S., Oztas D.Y., Gupta G. (2021). Atomistic simulations and in silico mutational profiling of protein stability and binding in the SARS-CoV-2 spike protein complexes with nanobodies: Molecular determinants of mutational escape mechanisms. ACS Omega.

[15] Chothia C., Lesk A.M. (1987). Canonical structures for the hypervariable regions of immunoglobulins. J. Mol. Biol..

[16] Zuo J., Li J., Zhang R., Xu L., Chen H., Jia X., Su Z., Zhao L., Huang X., Xie W. (2017). Institute collection and analysis of nanobodies (ican): A comprehensive database and analysis platform for nanobodies. BMC Genom..

[17] Berman H.M., Battistuz T., Bhat T.N., Bluhm W.F., Bourne P.E., Burkhardt K., Feng Z., Gilliland G.L., Iype L., Jain S. (2002). The protein data bank. Acta Crystallogr. Sect. D Biol. Crystallogr..

[18] Deszyński P., Młokosiewicz J., Volanakis A., Jaszczyszyn I., Castellana N., Bonissone S., Ganesan R., Krawczyk K. (2022). Indi-integrated nanobody database for immunoinformatics. Nucleic Acids Res..

[19] Noël F., Malpertuy A., de Brevern A.G. (2016). Global analysis of vhhs framework regions with a structural alphabet. Biochimie.

[20] Melarkode Vattekatte A., Shinada N.K., Narwani T.J., Noël F., Bertrand O., Meyniel J.P., Malpertuy A., Gelly J.C., Cadet F., de Brevern A.G. (2020). Discrete analysis of camelid variable domains: Sequences, structures, and in-silico structure prediction. PeerJ.

[21] Melarkode Vattekatte A., Cadet F., Gelly J.C., de Brevern A.G. (2021). Insights into comparative modeling of v(h)h domains. Int. J. Mol. Sci..

[22] Wang Y.T., Liao J.M., Chen C.L., Su Z.Y., Chen C.H., Hu J.J. (2008). Potential of mean force for human lysozyme–camelid vhh hl6 antibody interaction studies. Chem. Phys. Lett.

[23] Su Z.Y., Wang Y.T. (2009). A molecular dynamics simulation of the human lysozyme—Camelid vhh hl6 antibody system. Int. J. Mol. Sci..

[24] Velez-Vega C., Fenwick M.K., Escobedo F.A. (2009). Simulated mutagenesis of the hypervariable loops of a llama vhh domain for the recovery of canonical conformations. J. Phys. Chem. B.

[25] Soler M.A., de Marco A., Fortuna S. (2016). Molecular dynamics simulations and docking enable to explore the biophysical factors controlling the yields of engineered nanobodies. Sci. Rep..

[26] Mohseni A., Molakarimi M., Taghdir M., Sajedi R.H., Hasannia S. (2019). Exploring single-domain antibody thermostability by molecular dynamics simulation. J. Biomol. Struct. Dyn..

[27] Bekker G.J., Ma B., Kamiya N. (2019). Thermal stability of single-domain antibodies estimated by molecular dynamics simulations. Protein Sci. Publ. Protein Soc..

[28] Zabetakis D., Shriver-Lake L.C., Olson M.A., Goldman E.R., Anderson G.P. (2019). Experimental evaluation of single-domain antibodies predicted by molecular dynamics simulations to have elevated thermal stability. Protein Sci. Publ. Protein Soc..

[29] Soler M.A., Fortuna S., de Marco A., Laio A. (2018). Binding affinity prediction of nanobody-protein complexes by scoring of molecular dynamics trajectories. Phys. Chem. Chem. Phys..

[30] Ikeuchi E., Kuroda D., Nakakido M., Murakami A., Tsumoto K. (2021). Delicate balance among thermal stability, binding affinity, and conformational space explored by single-domain v(h)h antibodies. Sci. Rep..

[31] Fernández-Quintero M.L., DeRose E.F., Gabel S.A., Mueller G.A., Liedl K.R. (2022). Nanobody paratope ensembles in solution characterized by md simulations and nmr. Int. J. Mol. Sci..

[32] Gray E.R., Brookes J.C., Caillat C., Turbé V., Webb B.L.J., Granger L.A., Miller B.S., McCoy L.E., El Khattabi M., Verrips C.T. (2017). Unravelling the molecular basis of high affinity nanobodies against hiv p24: In vitro functional, structural, and in silico insights. ACS Infect. Dis..

[33] Murakami T., Kumachi S., Matsunaga Y., Sato M., Wakabayashi-Nakao K., Masaki H., Yonehara R., Motohashi M., Nemoto N., Tsuchiya M. (2022). Construction of a humanized artificial vhh library reproducing structural features of camelid vhhs for therapeutics. Antibodies.

[34] Lesne J., Chang H.J., De Visch A., Paloni M., Barthe P., Guichou J.F., Mayonove P., Barducci A., Labesse G., Bonnet J. (2019). Structural basis for chemically-induced homodimerization of a single domain antibody. Sci. Rep..

[35] Offmann B., Tyagi M., de Brevern A.G. (2007). Local protein structures. Curr. Bioinform..

[36] Craveur P., Joseph A.P., Esque J., Narwani T.J., Noël F., Shinada N., Goguet M., Leonard S., Poulain P., Bertrand O. (2015). Protein flexibility in the light of structural alphabets. Front. Mol. Biosci..

[37] Melarkode Vattekatte A., Narwani T.J., Floch A., Maljković M., Bisoo S., Shinada N.K., Kranjc A., Gelly J.C., Srinivasan N., Mitić N. (2020). A structural entropy index to analyse local conformations in intrinsically disordered proteins. J. Struct. Biol..

[38] De Brevern A.G. (2020). Analysis of protein disorder predictions in the light of a protein structural alphabet. Biomolecules.

[39] Sievers F., Wilm A., Dineen D., Gibson T.J., Karplus K., Li W., Lopez R., McWilliam H., Remmert M., Söding J. (2011). Fast, scalable generation of high-quality protein multiple sequence alignments using clustal omega. Mol. Syst. Biol..

[40] Mitchell L.S., Colwell L.J. (2018). Comparative analysis of nanobody sequence and structure data. Proteins.

[41] Kelow S.P., Adolf-Bryfogle J., Dunbrack R.L. (2020). Hiding in plain sight: Structure and sequence analysis reveals the importance of the antibody de loop for antibody-antigen binding. mAbs.

[42] North B., Lehmann A., Dunbrack R.L. (2011). A new clustering of antibody cdr loop conformations. J. Mol. Biol..

[43] de Brevern A.G., Etchebest C., Hazout S. (2000). Bayesian probabilistic approach for predicting backbone structures in terms of protein blocks. Proteins.

[44] Joseph A.P., Agarwal G., Mahajan S., Gelly J.C., Swapna L.S., Offmann B., Cadet F., Bornot A., Tyagi M., Valadié H. (2010). A short survey on protein blocks. Biophys. Rev..

[45] Bornot A., Etchebest C., de Brevern A.G. (2009). A new prediction strategy for long local protein structures using an original description. Proteins.

[46] Narwani T.J., Craveur P., Shinada N.K., Floch A., Santuz H., Vattekatte A.M., Srinivasan N., Rebehmed J., Gelly J.C., Etchebest C. (2020). Discrete analyses of protein dynamics. J. Biomol. Struct. Dyn..

[47] Goguet M., Narwani T.J., Petermann R., Jallu V., de Brevern A.G. (2017). In silico analysis of glanzmann variants of calf-1 domain of α(iib)β(3) integrin revealed dynamic allosteric effect. Sci. Rep..

[48] Anies S., Jallu V., Diharce J., Narwani T.J., de Brevern A.G. (2022). Analysis of integrin α(iib) subunit dynamics reveals long-range effects of missense mutations on calf domains. Int. J. Mol. Sci..

[49] Wesolowski J., Alzogaray V., Reyelt J., Unger M., Juarez K., Urrutia M., Cauerhff A., Danquah W., Rissiek B., Scheuplein F. (2009). Single domain antibodies: Promising experimental and therapeutic tools in infection and immunity. Med. Microbiol. Immunol..

[50] Bornot A., Etchebest C., de Brevern A.G. (2011). Predicting protein flexibility through the prediction of local structures. Proteins.

[51] De Brevern A.G., Bornot A., Craveur P., Etchebest C., Gelly J.C. (2012). Predyflexy: Flexibility and local structure prediction from sequence. Nucleic Acids Res..

[52] Narwani T.J., Etchebest C., Craveur P., Léonard S., Rebehmed J., Srinivasan N., Bornot A., Gelly J.C., de Brevern A.G. (2019). In silico prediction of protein flexibility with local structure approach. Biochimie.

[53] Van Der Spoel D., Lindahl E., Hess B., Groenhof G., Mark A.E., Berendsen H.J. (2005). Gromacs: Fast, flexible, and free. J. Comput. Chem..

[54] Rakhshani H., Dehghanian E., Rahati A. (2019). Enhanced gromacs: Toward a better numerical simulation framework. J. Mol. Model..

[55] Lindorff-Larsen K., Piana S., Palmo K., Maragakis P., Klepeis J.L., Dror R.O., Shaw D.E. (2010). Improved side-chain torsion potentials for the amber ff99sb protein force field. Proteins.

[56] Barnoud J., Santuz H., Craveur P., Joseph A.P., Jallu V., de Brevern A.G., Poulain P. (2017). Pbxplore: A tool to analyze local protein structure and deformability with protein blocks. PeerJ.

[57] Faure G., Joseph A.P., Craveur P., Narwani T.J., Srinivasan N., Gelly J.C., Rebehmed J., de Brevern A.G. (2019). Ipbavizu: A pymol plugin for an efficient 3d protein structure superimposition approach. Source Code Biol. Med..

[58] Joseph A.P., Srinivasan N., de Brevern A.G. (2011). Improvement of protein structure comparison using a structural alphabet. Biochimie.

[59] Léonard S., Joseph A.P., Srinivasan N., Gelly J.C., de Brevern A.G. (2014). Mulpba: An efficient multiple protein structure alignment method based on a structural alphabet. J. Biomol. Struct. Dyn..

[60] DeLano W.L. (2002). Pymol.

[61] Delano W.L. (2013). The Pymol Molecular Graphics System on World Wide Web. http://www.pymol.org.

[62] Humphrey W., Dalke A., Schulten K. (1996). Vmd: Visual molecular dynamics. J. Mol. Graph..

[63] Kabsch W., Sander C. (1983). Dictionary of protein secondary structure: Pattern recognition of hydrogen-bonded and geometrical features. Biopolymers.

[64] Smith D.K., Radivojac P., Obradovic Z., Dunker A.K., Zhu G. (2003). Improved amino acid flexibility parameters. Protein Sci. Publ. Protein Soc..

[65] Celton M., Malpertuy A., Lelandais G., de Brevern A.G. (2010). Comparative analysis of missing value imputation methods to improve clustering and interpretation of microarray experiments. BMC Genom..

[66] De Brevern A.G., Hazout S., Malpertuy A. (2004). Influence of microarrays experiments missing values on the stability of gene groups by hierarchical clustering. BMC Bioinform..

[67] Harris C.R., Millman K.J., van der Walt S.J., Gommers R., Virtanen P., Cournapeau D., Wieser E., Taylor J., Berg S., Smith N.J. (2020). Array programming with numpy. Nature.

[68] Team R.D.C. (2011). R: A Language and Environment for Statistical Computing.

[69] Waterhouse A.M., Procter J.B., Martin D.M., Clamp M., Barton G.J. (2009). Jalview version 2—A multiple sequence alignment editor and analysis workbench. Bioinformatics.

