# Supplementary material for: General Trends of the Camelidae Antibody VHHs Domain Dynamics
Source: Int J Mol Sci. 2023 Feb 24;24(5):4511. doi: 10.3390/ijms24054511 (PMC10003728; doi:10.3390/ijms24054511)
Supplement: Supplementary file 1 [file ijms-24-04511-s001.zip › ijms-2242392-supplementary.pdf]

Title: General trends of the V_HHs domain dynamics.

Authors: Akhila Melarkode Vattekatte*, Julien Diharce, Joseph Rebehmed, Frédéric Cadet, Fabrice Gardebien, Catherine Etchebest & Alexandre G. de Brevern*

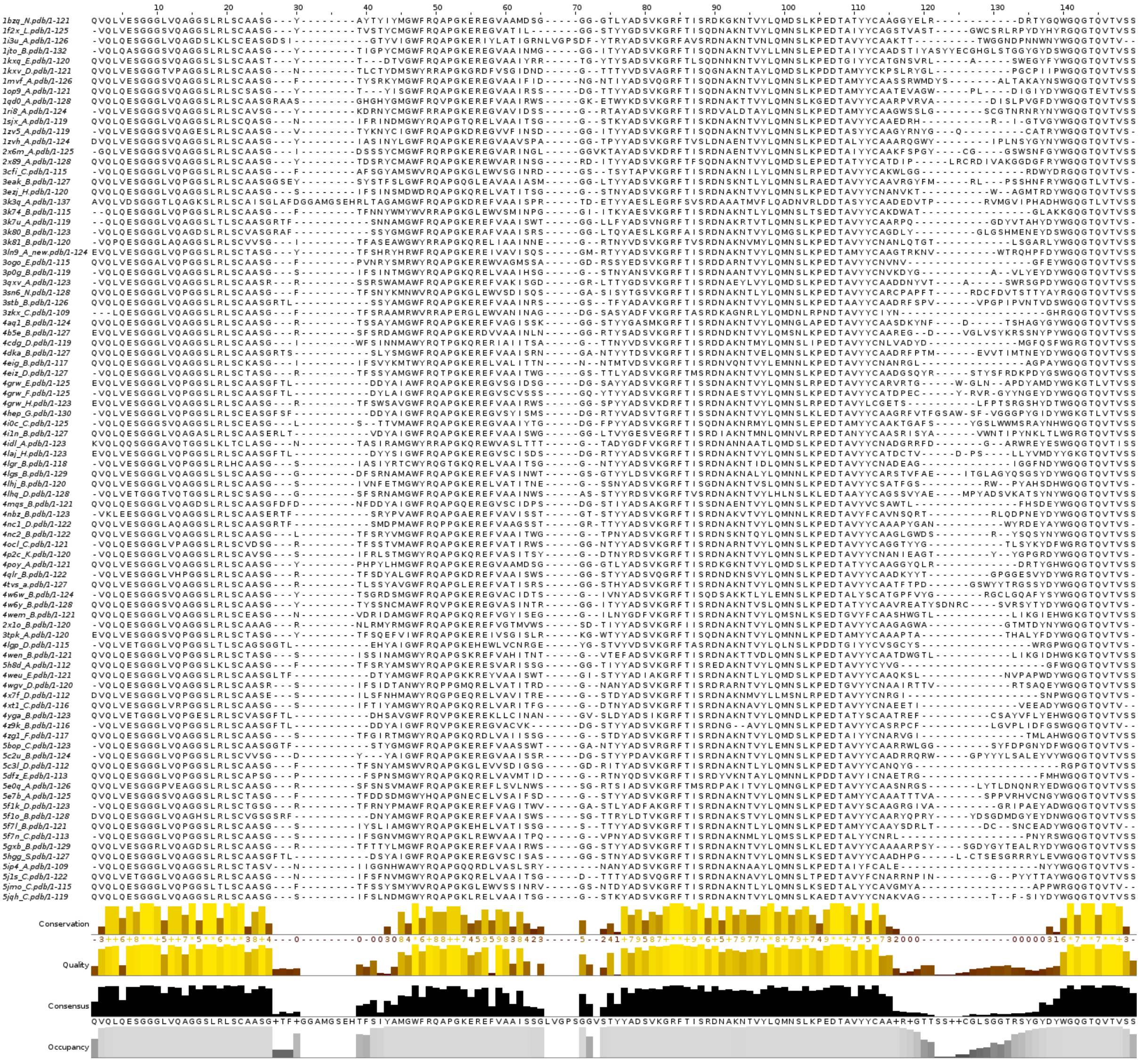

Figure S1. Multiple sequence alignment of VHH sequences. Sequences are extracted from PDB entries in the dataset and aligned with ClustalOmega. The Multiple Sequence Alignment was visualised with Jalview [1]. The conservation, quality and consensus of the residue of information at each position are also shown at the bottom of the alignment.

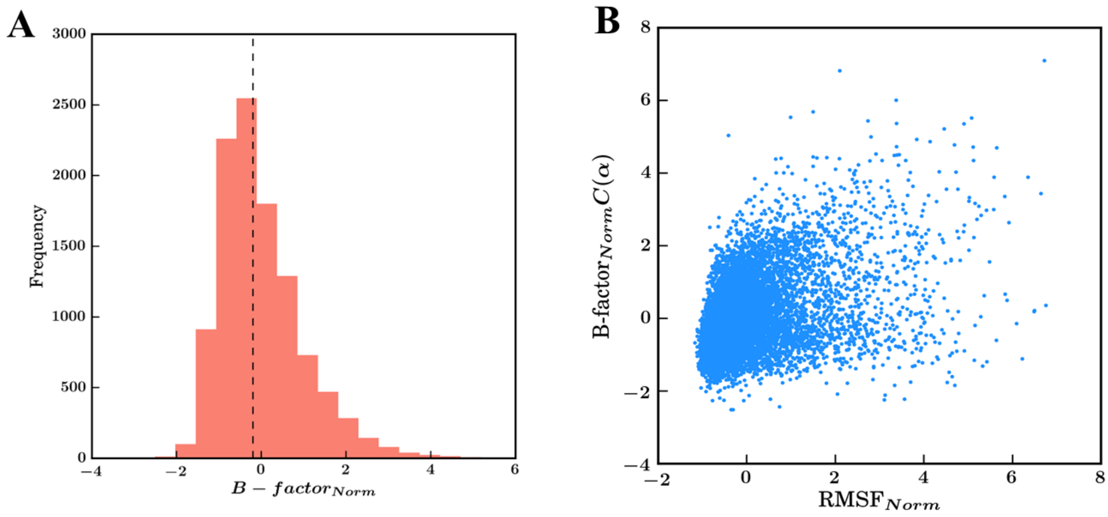

Figure S2. Distribution of flexibility metrics in VHH structures and MD simulations. Conformational diversity in dataset was calculated using normalized Cα B-factors from 88 VHH structures. (A) Distribution of normalized Cα B-factors (dashed line is the median value), and, (B) normalized Cα RMSF *vs.* normalized Cα B-factors.

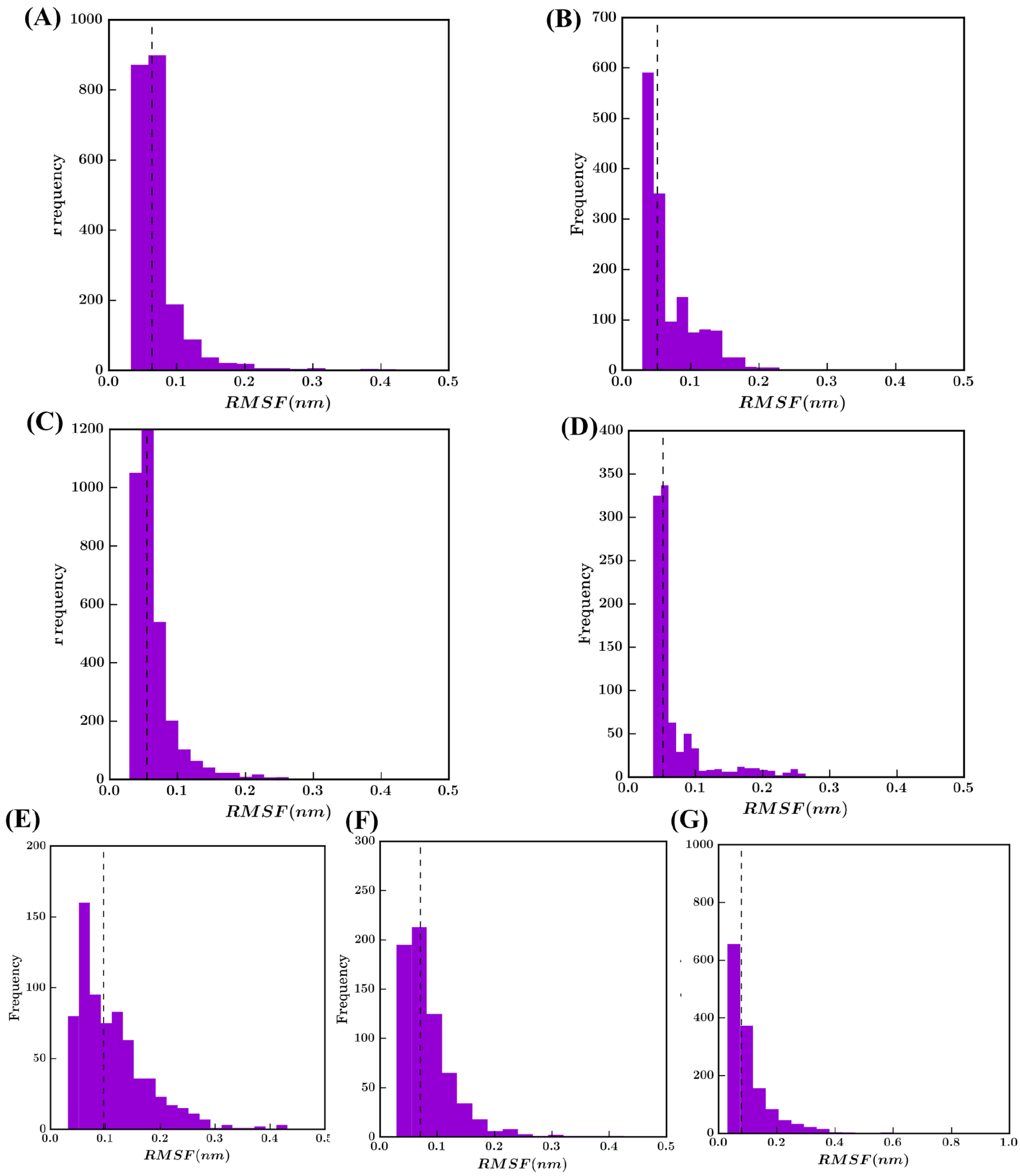

Figure S3. Distribution of normalized Cα RMSF values for the different VHH regions. (A) FR1, (B) FR2, (C) FR3, (D) FR4, (E) CDR1, (F) CDR2 and (G) CDR3.

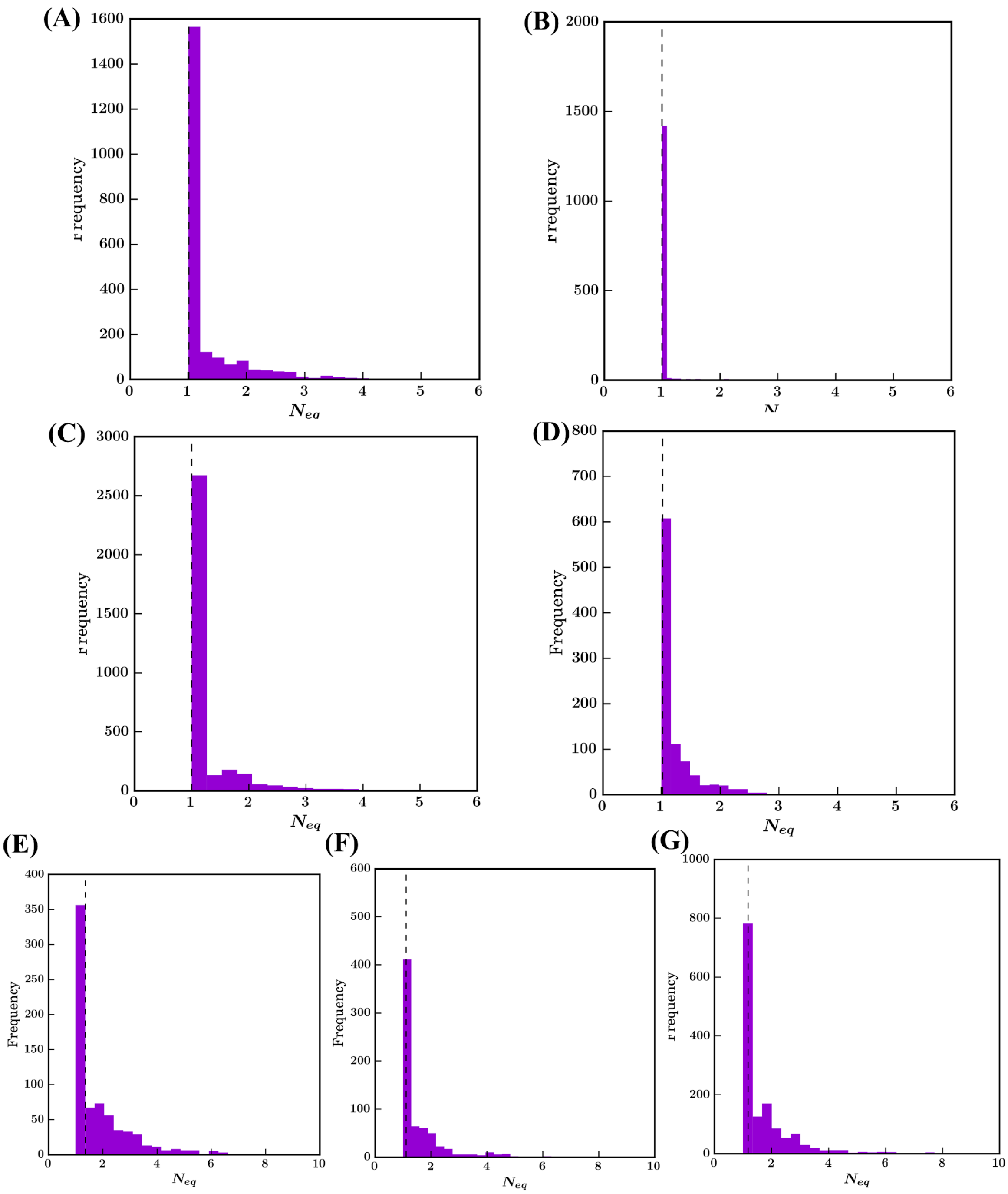

Figure S4. Distribution of N_{eq} values for the different V_HH regions. (A) FR1, (B) FR2, (C) FR3, (D) FR4, (E) CDR1, (F) CDR2 and (G) CDR3.

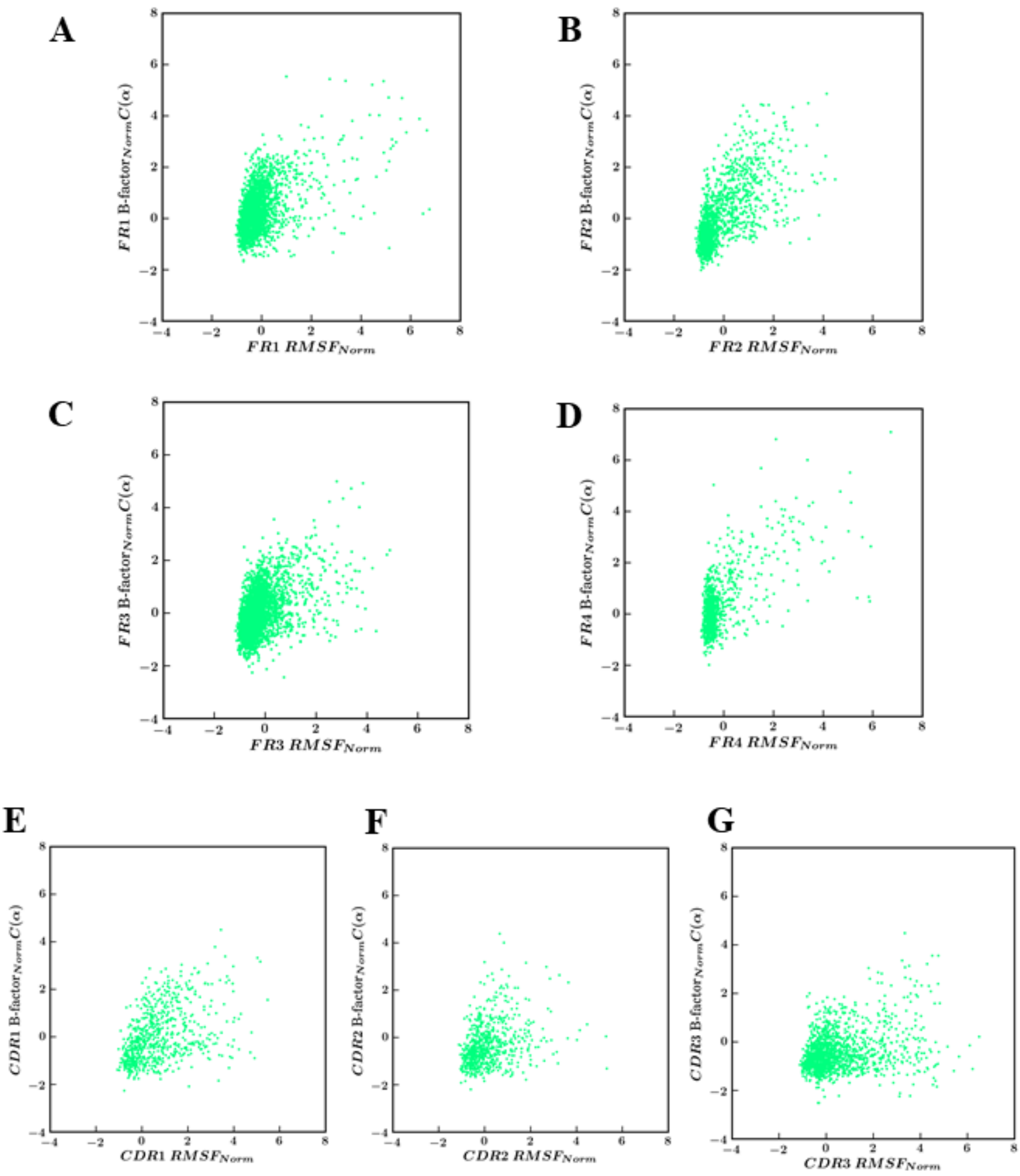

Figure S5. Correlation between normalized Cα B-factors (VHH structures) and normalized Cα RMSF (VHH trajectories) per region. (A) FR1, (B) FR2, (C) FR3, (D) FR4, (E) CDR1, (F) CDR2 and (G) CDR3.

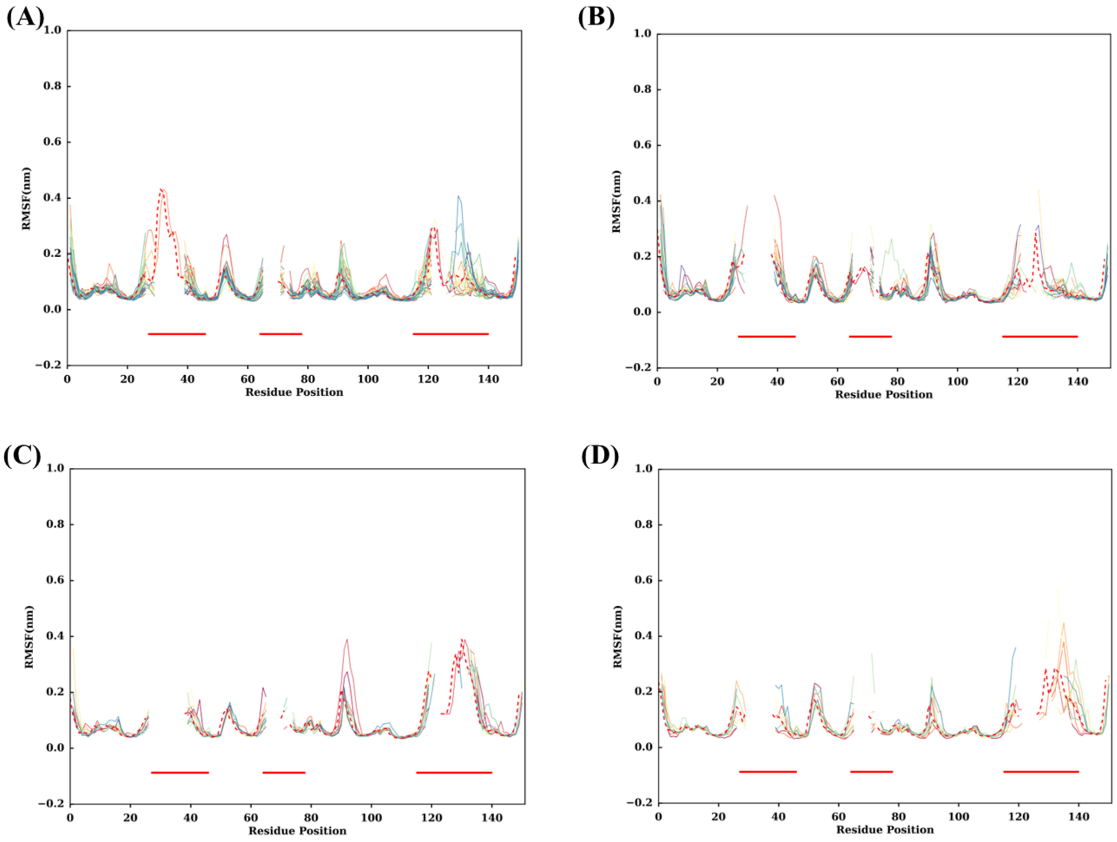

Figure S6. Cα RMSF values along the sequences for the different RMSF clusters. (A) RMSF cluster 1, (B) RMSF cluster 2, (C) RMSF cluster 3 and (D) RMSF cluster 4 values are shown. The mean values are shown with a red dotted line, and the 3 CDR locations are shown using red bars. Positions are taken from the multiple sequence alignment (see Figure S1).

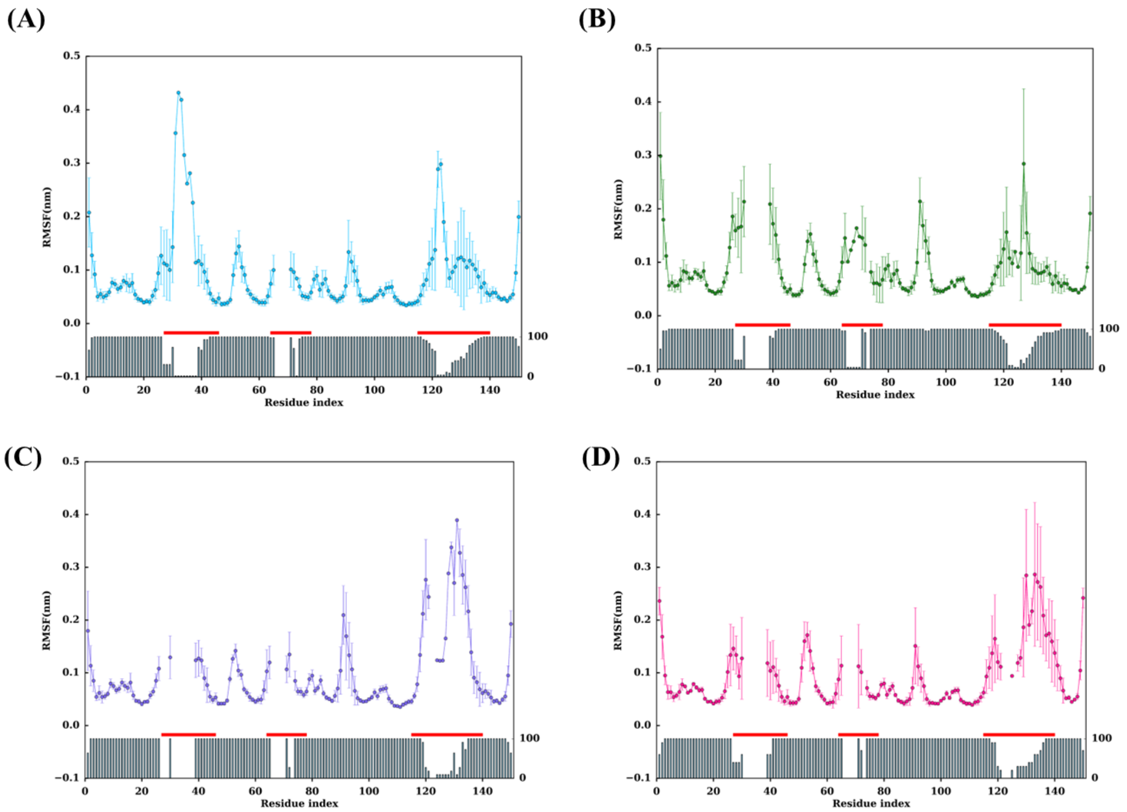

Figure S7. Mean and standard deviation C$_\alpha$ RMSF values of RMSF clusters. (A) RMSF cluster 1, (B) RMSF cluster 2, (C) RMSF cluster 3 and (D) RMSF cluster 4. The 3 CDRs are highlighted as red bars. Relative occurrence of amino acid is provided at the bottom of each graph as a histogram; the reference is the multiple sequence alignment (see Figure S1).

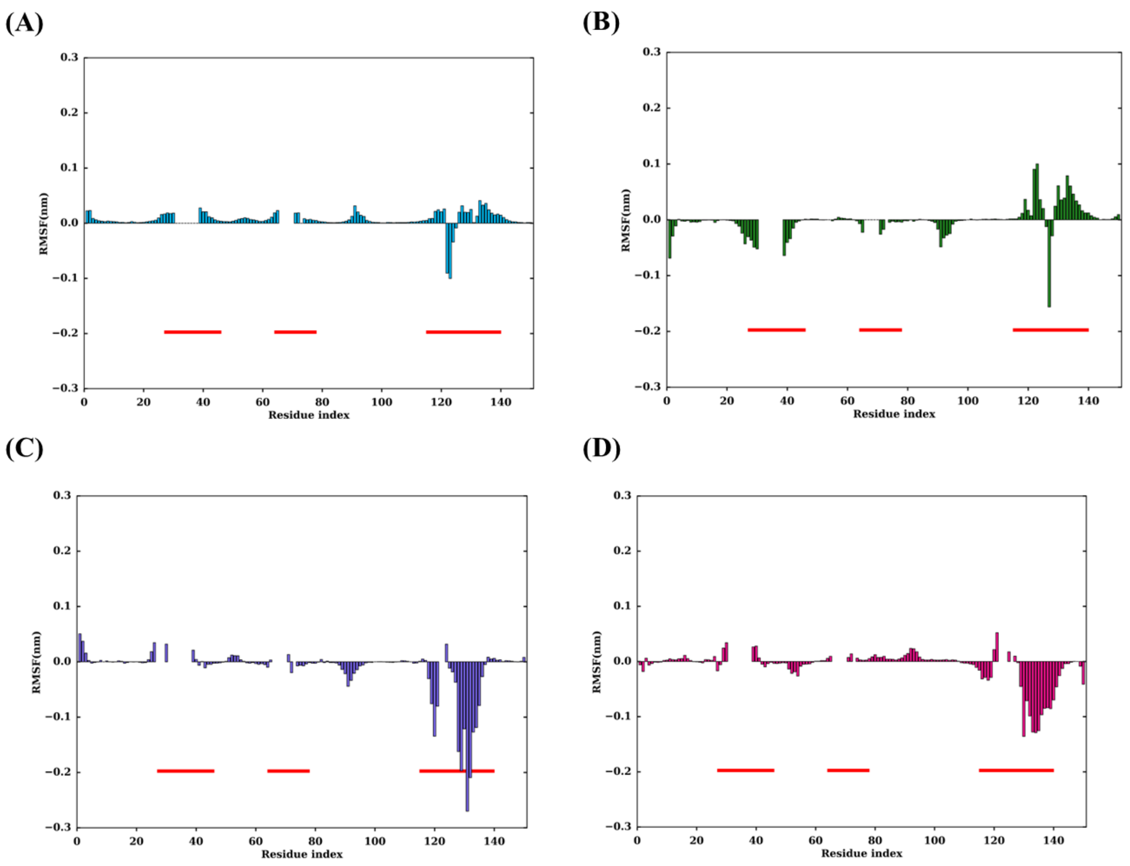

Figure S8. Differences between the general mean Cα RMSF values and the RMSF clusters. Is provided the difference between Cα RMSF mean values of all V_HHs (see Figure 3) and (A) of RMSF cluster 1, (B) of RMSF cluster 2, (C) of RMSF cluster 3, and (D) of RMSF cluster 4.

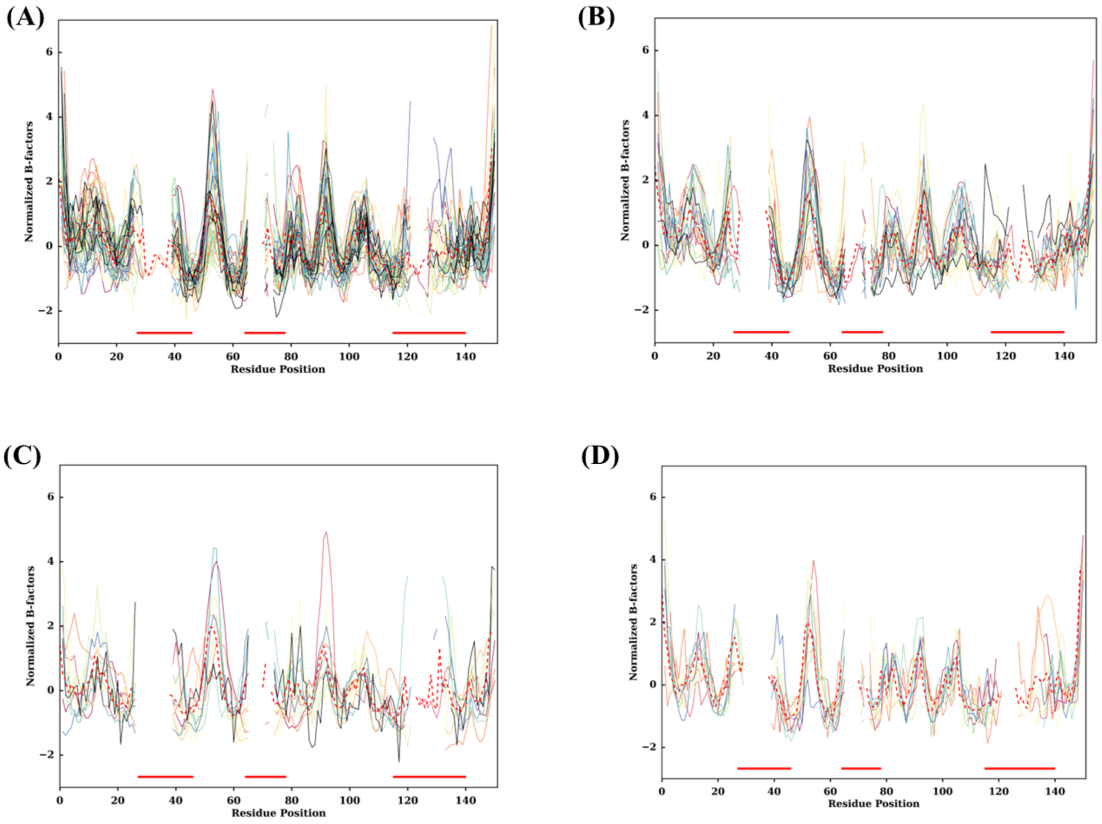

Figure S9. Normalized Cα B-factor values of RMSF clusters. (A) RMSF cluster 1, (B) RMSF cluster 2, (C) RMSF cluster 3 and (D) RMSF cluster 4 values are shown. The mean values are shown with a red dotted line and the 3 CDRs are demarcated using red bars.

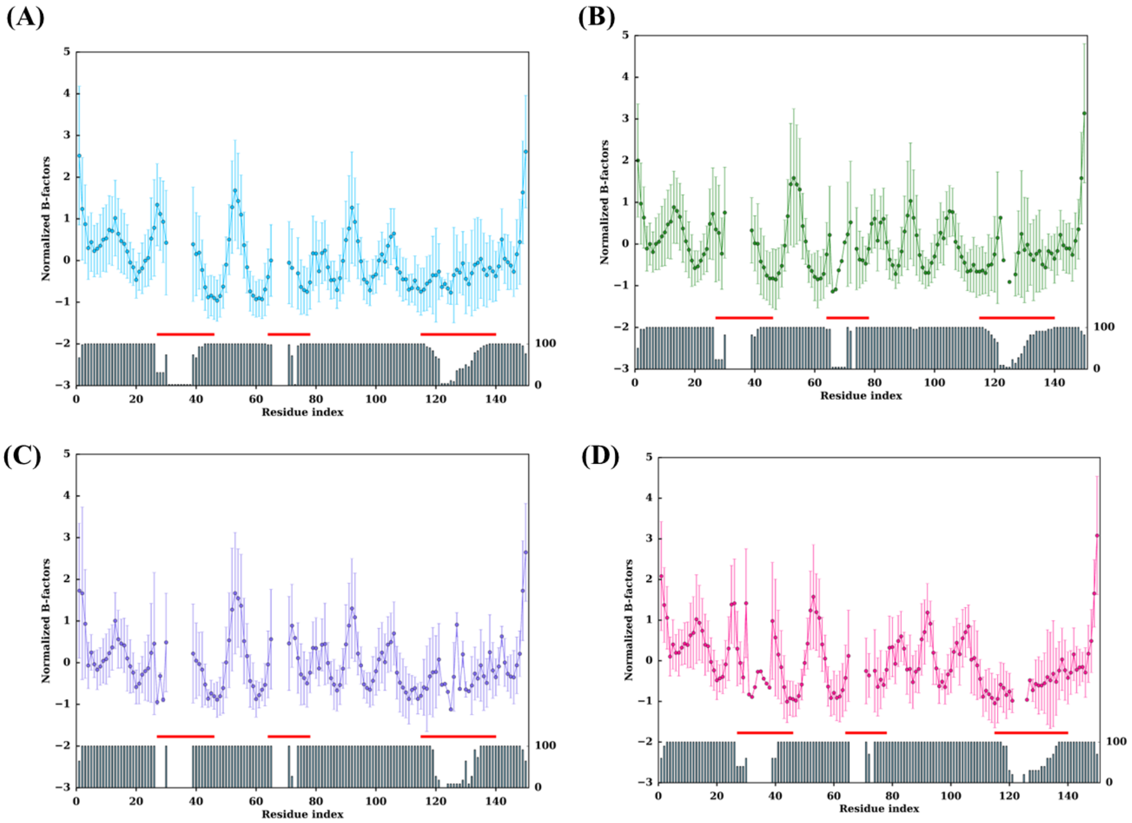

Figure S10. Mean and standard deviation normalized Cα B-factor values of RMSF clusters. (A) RMSF cluster 1, (B) RMSF cluster 2, (C) RMSF cluster 3 and (D) RMSF cluster 4. The 3 CDRs are highlighted as red bars. Relative occurrence of amino acid is provided at the bottom of each graph as a histogram, as the reference is the multiple sequence alignment (see Figure S1).

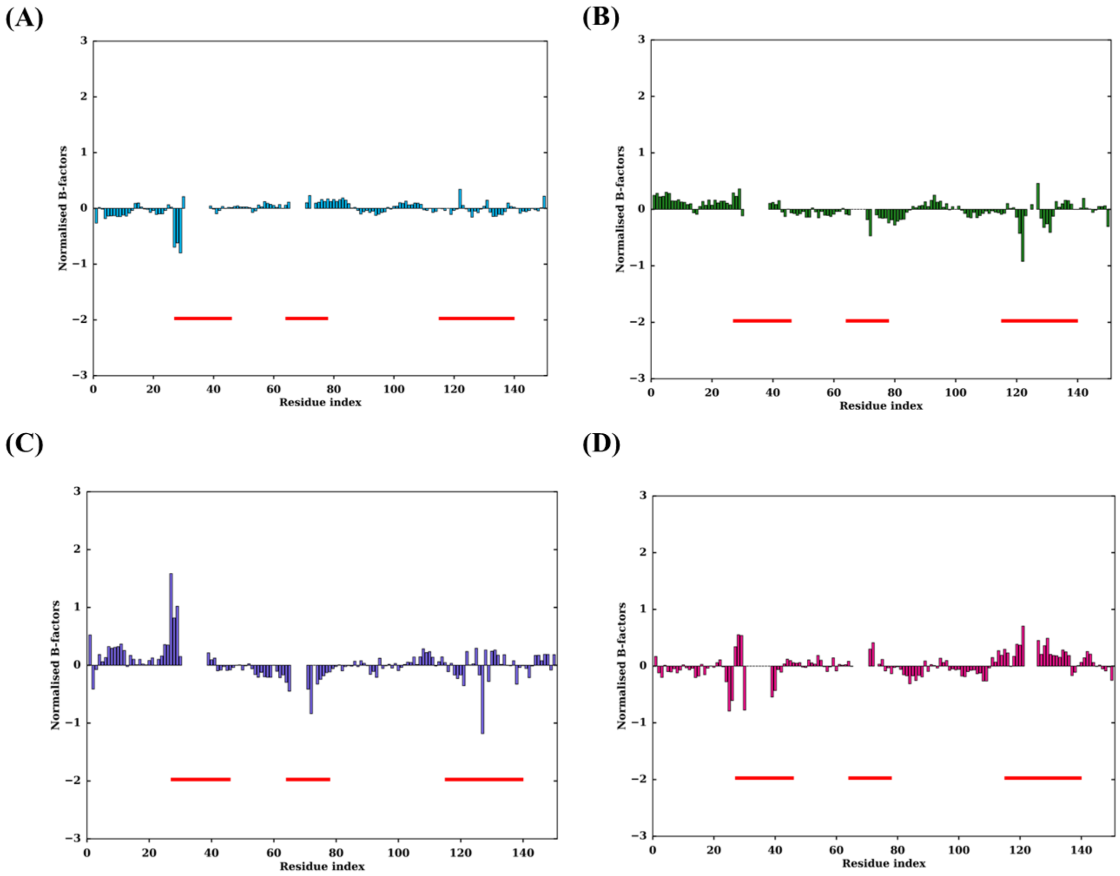

Figure S11. Analyses of RMSF cluster's normalized C_α B-factors. Is provided the difference between normalized C_α B-factor mean values of all V_HHs (see Figure 3) and (A) of RMSF cluster 1, (B) of RMSF cluster 2, (C) of RMSF cluster 3, and (D) of RMSF cluster 4.

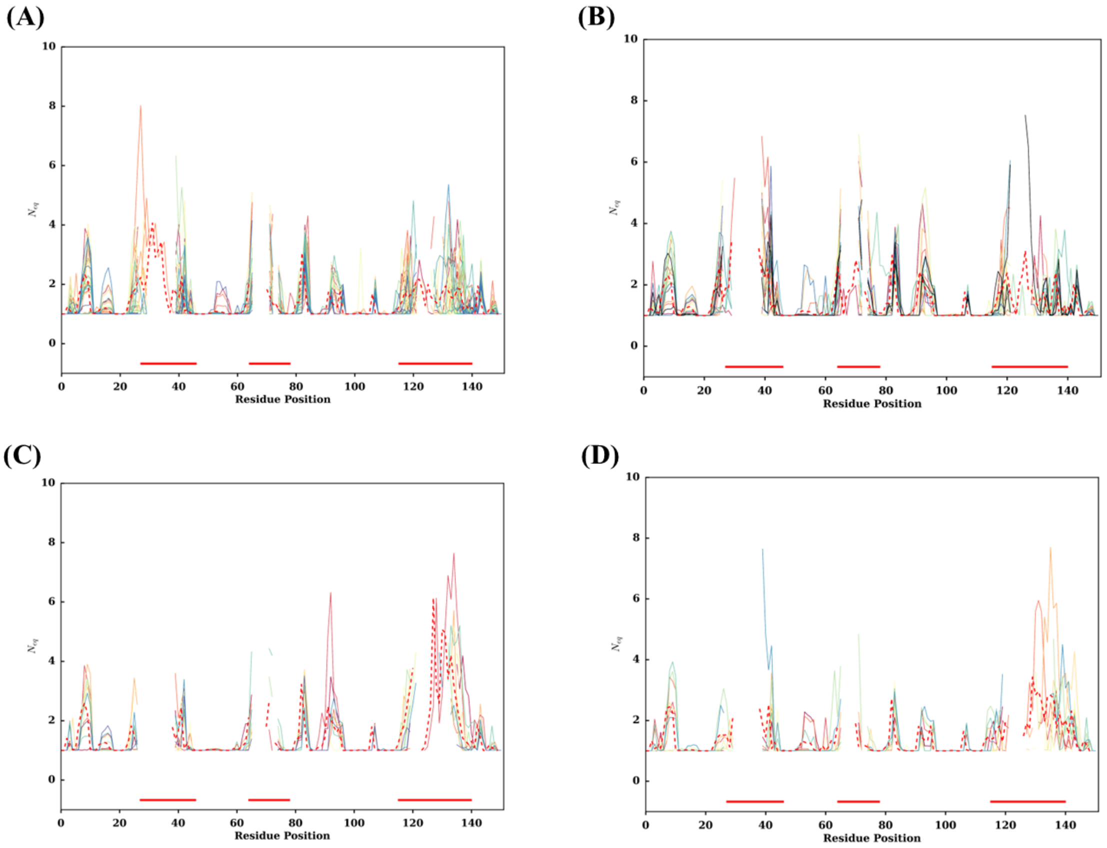

Figure S12. N_{eq} values of RMSF clusters. N_{eq} values of (A) RMSF cluster 1, (B) RMSF cluster 2, (C) RMSF cluster 3 and (D) RMSF cluster 4 are shown. The mean values are shown with a red dotted line and the 3 CDRs are demarcated using red bars.

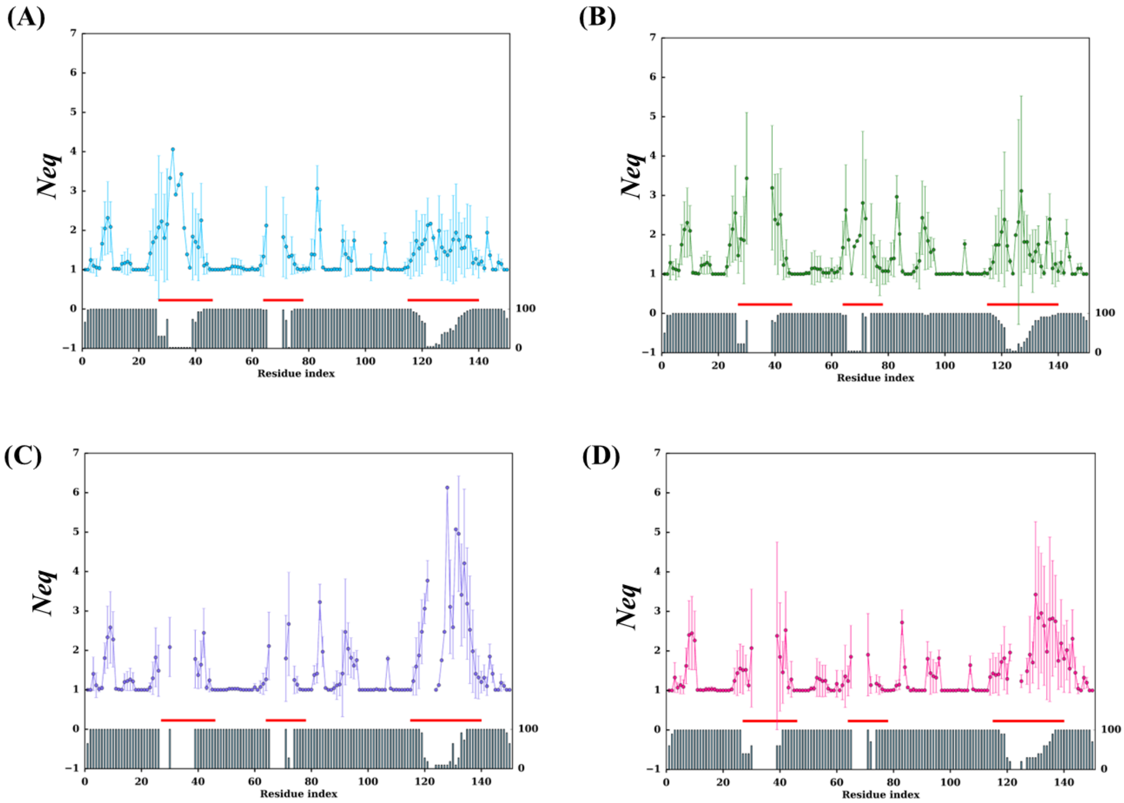

Figure S13. Mean and standard deviation N_{eq} values of RMSF clusters. The mean and standard deviation N_{eq} values of (A) RMSF cluster 1, (B) RMSF cluster 2, (C) RMSF cluster 3 and (D) RMSF cluster 4 are shown. The 3 CDRs are highlighted as red bars. Relative occurrence of amino acid is provided at the bottom of each graph as a histogram, as the reference is the multiple sequence alignment (see Figure S1).

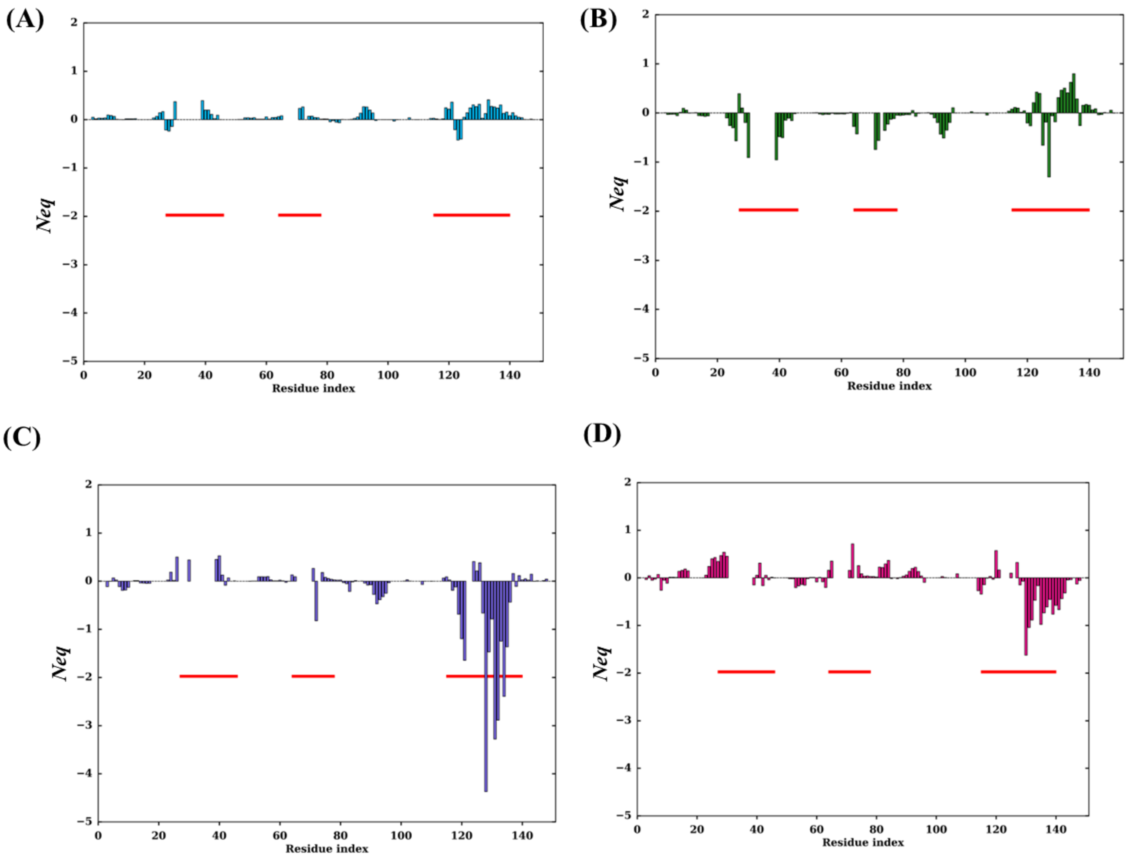

Figure S14. Analyses of N_{eq} cluster's RMSF. Is provided the difference between normalized N_{eq} mean values of all V_{H}Hs (see Figure 3) and (A) of RMSF cluster 1, (B) of RMSF cluster 2, (C) of RMSF cluster 3, and (D) of RMSF cluster 4.

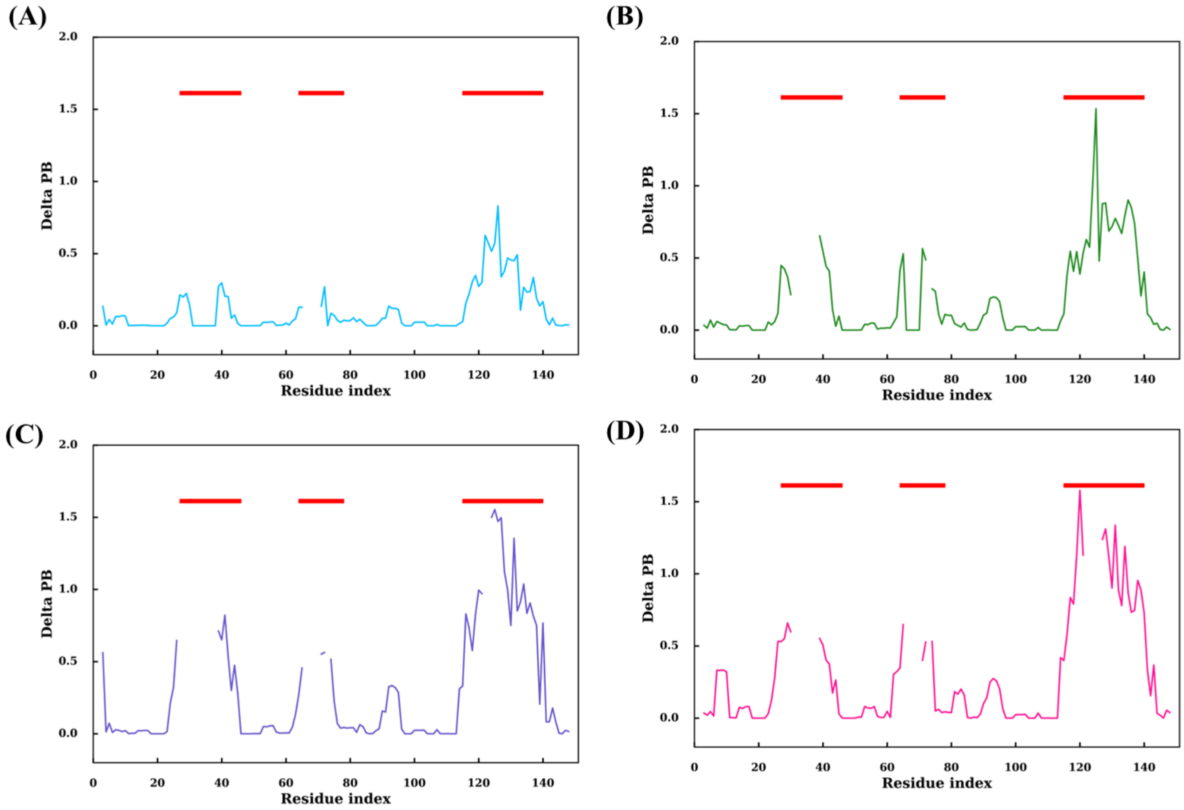

Figure S15. Specificity of PB signatures of RMSF clusters. ΔPB between all VHHs and (A) RMSF cluster 1, (B) RMSF cluster 2, (C) RMSF cluster 3 and (D) RMSF cluster 4.

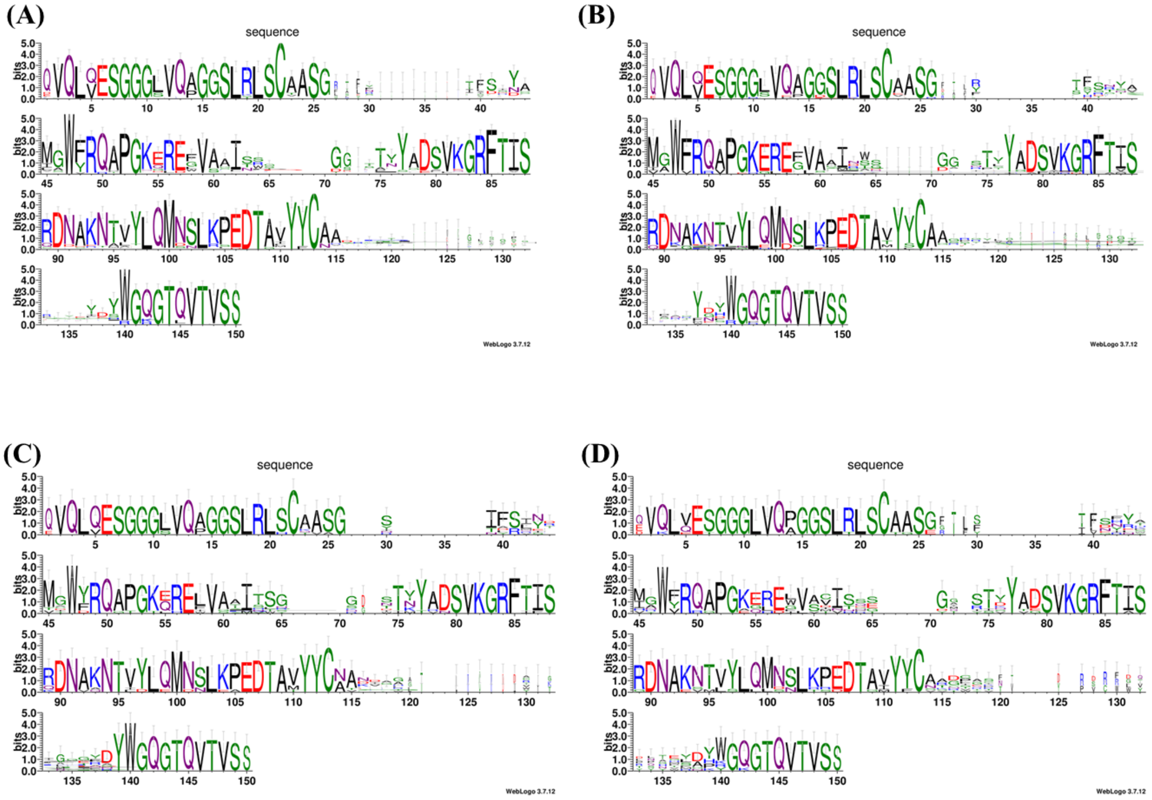

Figure S16. Amino acid conservation of VHH structures representing each RMSF cluster. Sequence logo representation of VHH sequences in each RMSF cluster with (A) RMSF cluster 1, (B) RMSF cluster 2, (C) RMSF cluster 3, and (D) RMSF cluster 4.

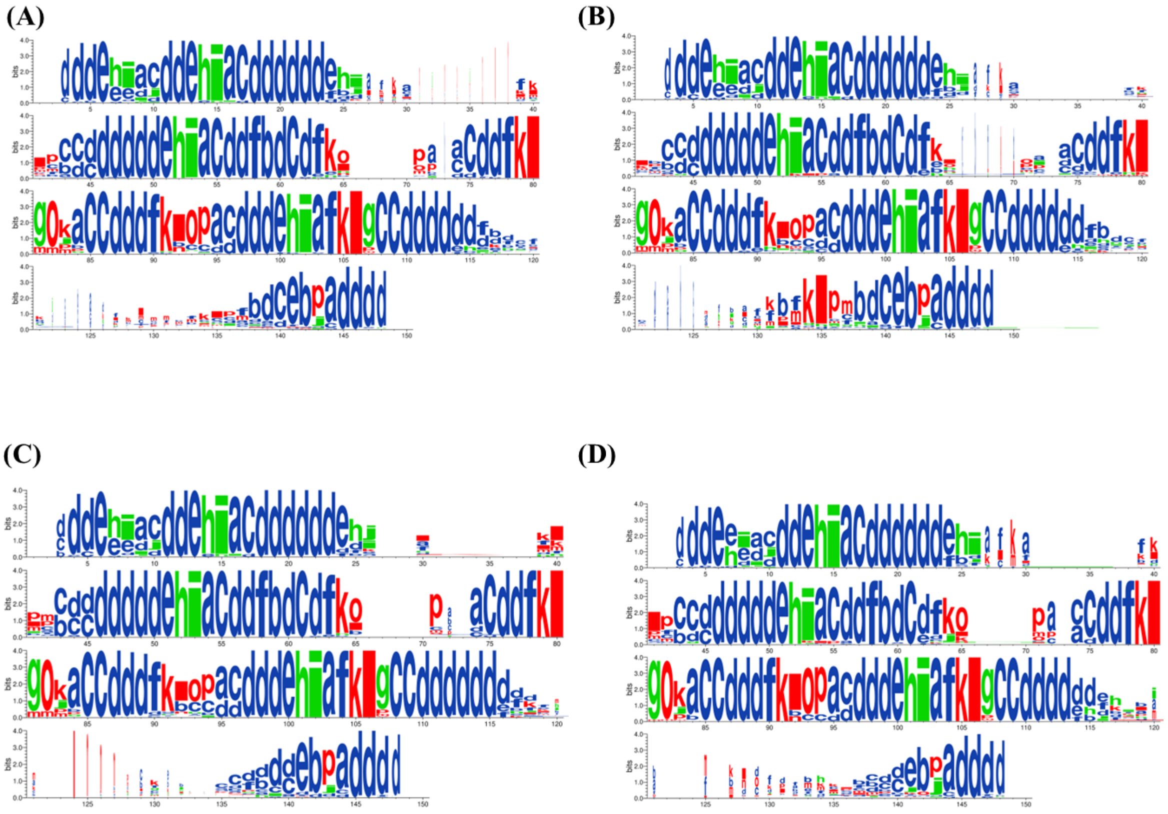

Figure S17. Protein Block conservation of VHH structures belonging to each RMSF cluster. Sequence logo representation of Protein Blocks assigned to RMSF clusters with (A) RMSF cluster 1, (B) RMSF cluster 2, (C) RMSF cluster 3 and (D) RMSF cluster 4.

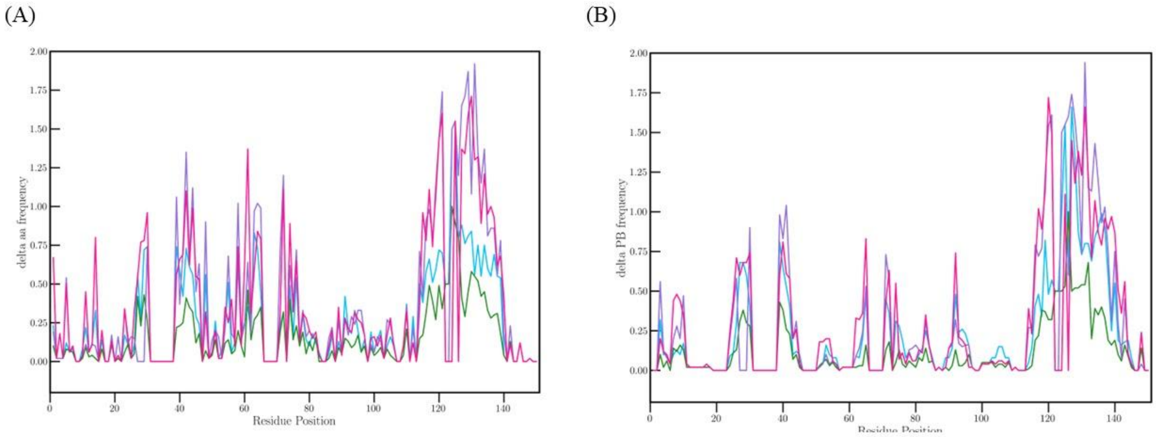

Figure S18. RMSF clusters' ΔAA and ΔPB between the clusters and the complete databank. (A) ΔAA of each of four clusters, and (B) ΔPB of each of four clusters. Cluster 1 shown in sky blue colour, cluster2 in forest green, cluster 3 in purple and cluster4 in pink.

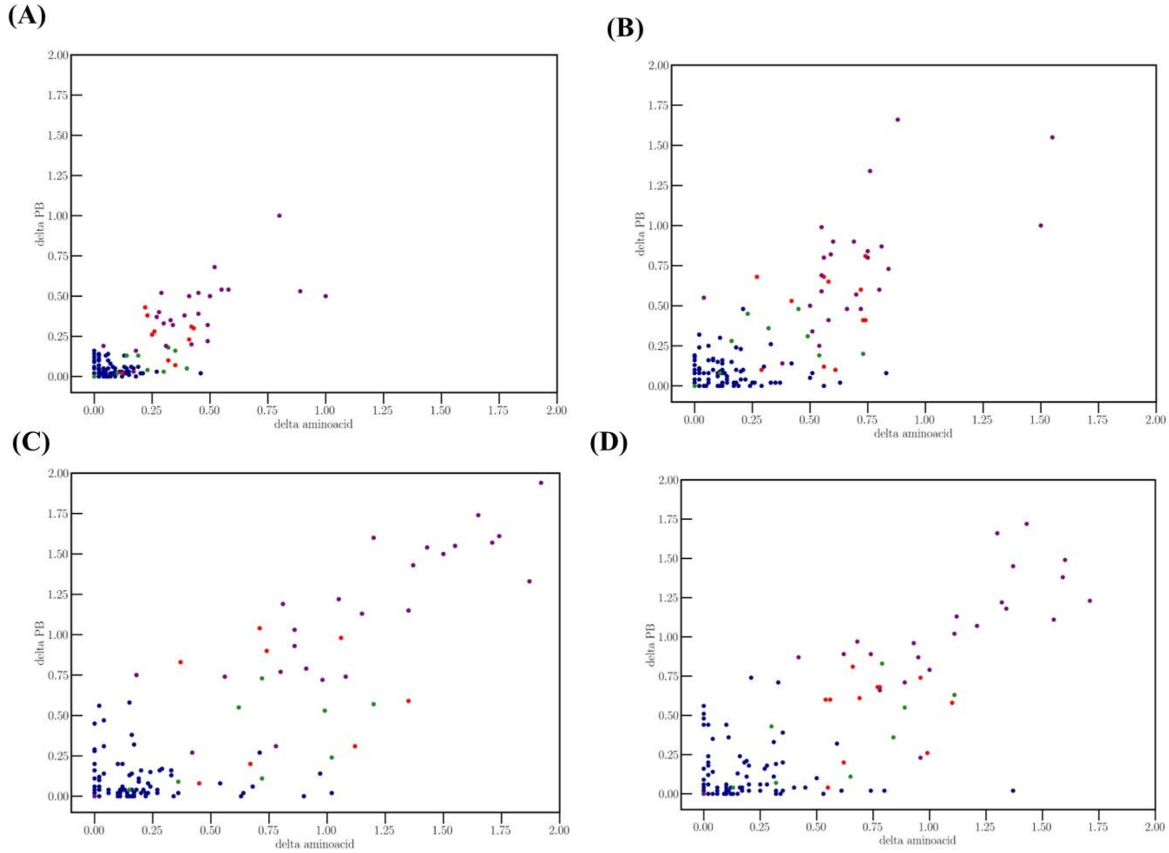

Figure S19. Correlation between ΔAA and ΔPB in different regions in the four clusters. (A) Cluster 1, (B) Cluster 2, (C) Cluster 3, and (D) Cluster 4. The FRs in each plot are shown in blue, CDR 1 in orange, CDR2 in green and CDR3 in purple. The x-axis in each figure is ΔAA and y-axis is ΔPB values.

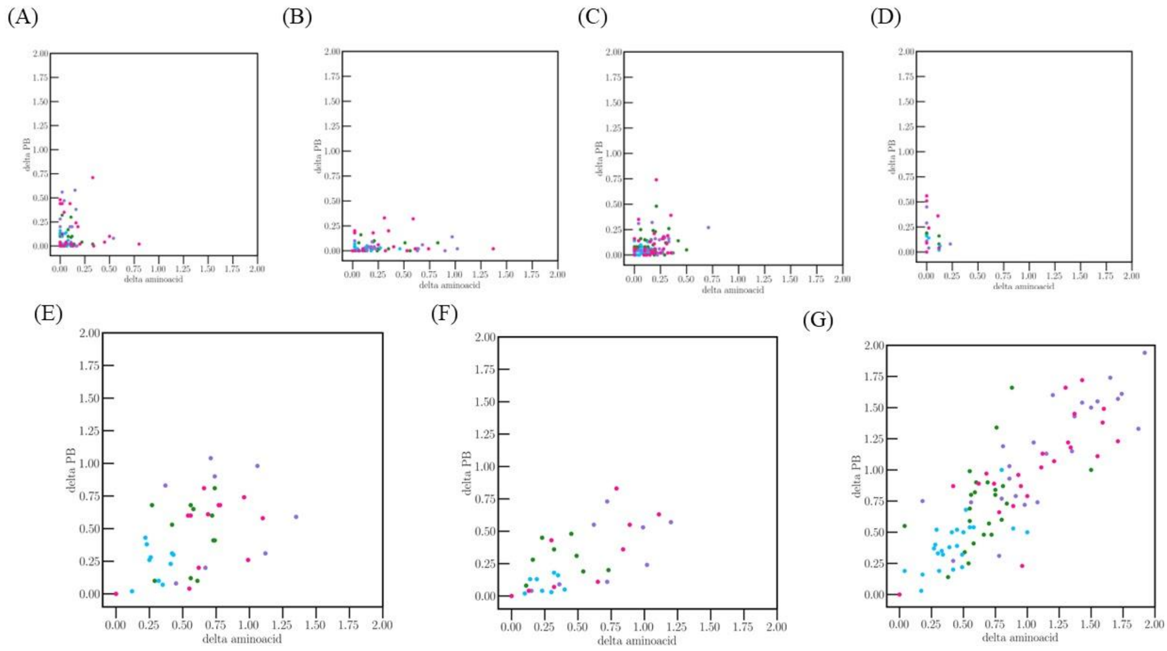

Figure S20. Correlation between ΔAA and ΔPB in different regions. (A) FR1, (B) FR2, (C) FR3), (D) FR4, (E) CDR1, (F) CDR2, and (G) CDR3. Colours are similar to Figure S18.

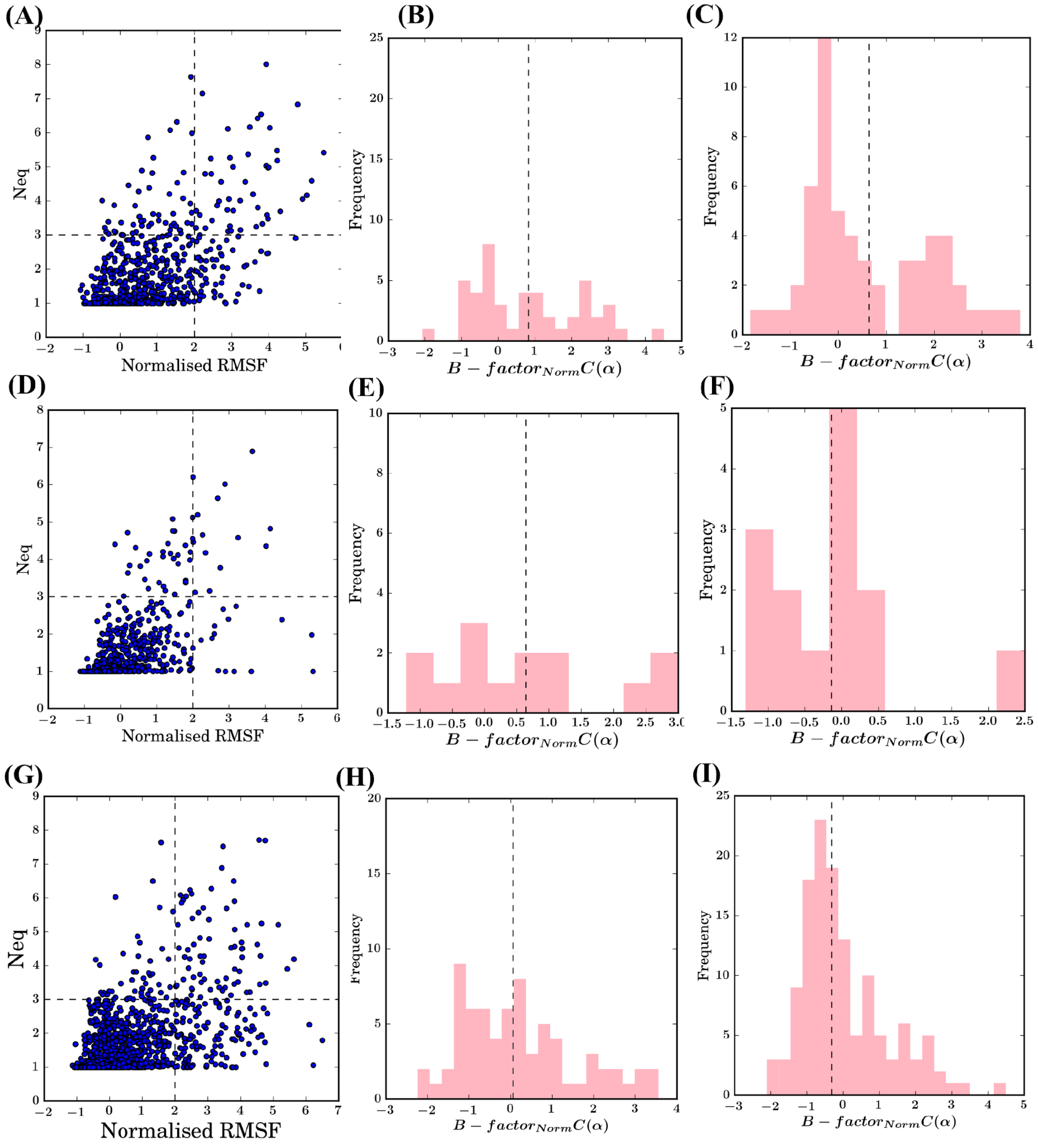

Figure S21. CDR flexibility. (A) to (C) is CDR1, (D) to F is CDR2 and (G) to (I) is CDR3. (A), (D) and (G) show the normalised RMSF vs. N_{eq}, while (B), (E) and (H) are the normalized C_α B-factors of flexible quadrant (nRMSF > 2 and N_{eq} > 3), and (C), (F) and (I) for mobile quadrant (nRMSF > 2 and N_{eq} < 3). The dotted line in each B-factor distribution represents the median value.

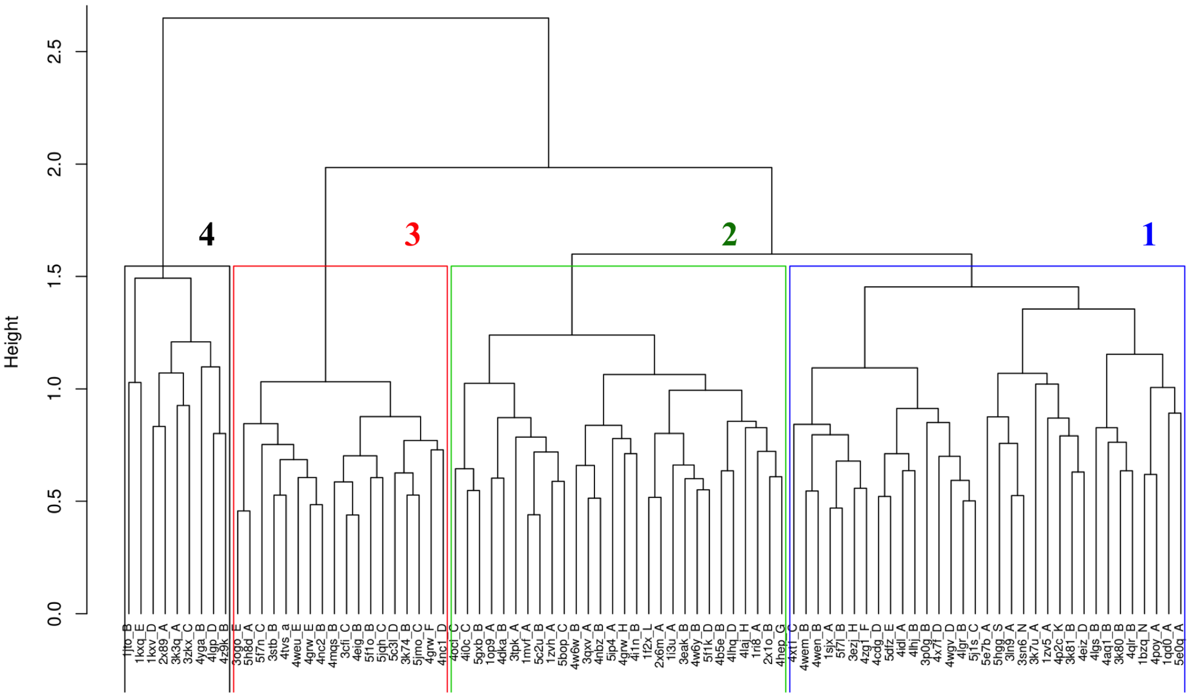

Figure S22. Hierarchical clustering based on ΔPB values. The different clusters are demarcated using different coloured boxes, with ΔPB cluster 1 (blue colour with 33 VHHs, i.e. 37.5%), ΔPB cluster 2 (green colour with 28 VHHs, i.e. 31.8%), ΔPB cluster 3 (red colour with 18 VHHs, i.e. 20.5%) and ΔPB cluster 4 (black colour with 9 VHHs, i.e. 10.2%).

Method S1. *Molecular Dynamics Analysis*

The analysis of MD trajectories is done using classic tools, such as the Root-Mean-Square Fluctuation (RMSF) of the Cα atoms using scripts from GROMACS software, and other more innovative approaches such as PBxplore [2] available on GitHub (https://pypi.org/project/pbxplore/). PBxplore allows it to assign Protein Blocks (see below) throughout the MD trajectories.

The RMSF is a calculation similar to the RMSD. Instead of computing positional differences between entire structures over time, it calculates how much a particular residue fluctuates from its average position during a simulation and thus obtains an estimate of its flexibility, with higher RMSF values assumed to indicate greater flexibility during the MD simulation.

Protein Blocks (PBs) are a structural alphabet composed of 16 local prototypes [3]. Each specific PB is characterized by the ϕ, ψ dihedral angles of five consecutive residues, with each PB assignment, focused on the central residue. Obtained through an unsupervised training approach and performed on a representative non-redundant databank, PBs give a reasonable approximation of all local protein 3D structures [4]. PBs are very efficient in tasks such as protein superimpositions [5-7] and MD analyses [8], even for disorder proteins [9]. They are labelled from *a* to *p*: PBs *m* and *d* can be roughly described as prototypes for α-helix and central β-strand, respectively. PBs *a* to *c* primarily represent β-strand N-caps, and PBs *e* and *f* represent β-strand C-caps; PBs, *a* to *j*, are specific to coils; PBs *k* and *l* to α-helix N-caps while PBs *n* to *p* to α-helix C-caps. PB assignment was carried out using our PBxplore tool [2].

RMSF cluster 1	FR1	CDR1	FR2	CDR2	FR3	CDR3	FR4
B-factor	0	--- / 0	0	0	0	0	0
RMSF	0	+	0	0 / +	0	----	0
*N*eq	0	++	0	0	+	++	0
RMSF cluster 2	FR1	CDR1	FR2	CDR2	FR3	CDR3	FR4
B-factor	0	0	0	-	0	---	0
RMSF	-- / 0	--	0	0 / -	--	+++/----/+++	0
*N*eq	0	---	0	--	--	+++/----/+++	0
RMSF cluster 3	FR1	CDR1	FR2	CDR2	FR3	CDR3	FR4
B-factor	+++	++++ / 0	0	----	0	----	0
RMSF	++ / 0	0	0	0	--	----	0
*N*eq	0	+	0	---	--	----	0
RMSF cluster 4	FR1	CDR1	FR2	CDR2	FR3	CDR3	FR4
B-factor	0	--/++/--	0	0 / ++	0	++++	0
RMSF	0	0	0	0	0	----	+
*N*eq	0	++	0	++	0	----	0
+: more flexibility							
-: more rigidity							
when a region is split into two consecutive tendencies, they are written with a '/'							

Table S1. Region wise trends in V_HH dynamics in each cluster. Is shown for every RMSF cluster the tendencies observed for each CDR and FR through normalized C_α B-factor, RMSF and N_{eq}.

RMSF clusters / PB clusters	1	2	3	4
1	9	7	1	5
2	13	14	6	9
3	3	4	0	4
4	5	3	2	0
Outlier 1	1	0	0	0
Outlier 2	1	0	0	0
Outlier 3	1	0	0	0

Table S2. Confusion matrix between RMSF hierarchical clustering and ΔPB hierarchical clustering.

References

1. Waterhouse, A.M.; Procter, J.B.; Martin, D.M.; Clamp, M.; Barton, G.J. Jalview version 2--a multiple sequence alignment editor and analysis workbench. *Bioinformatics (Oxford, England)* **2009**, *25*, 1189-1191.
2. Barnoud, J.; Santuz, H.; Craveur, P.; Joseph, A.P.; Jallu, V.; de Brevern, A.G.; Poulain, P. Pbxplore: A tool to analyze local protein structure and deformability with protein blocks. *PeerJ* **2017**, *5*, e4013.
3. de Brevern, A.G.; Etchebest, C.; Hazout, S. Bayesian probabilistic approach for predicting backbone structures in terms of protein blocks. *Proteins* **2000**, *41*, 271-287.
4. Joseph, A.P.; Agarwal, G.; Mahajan, S.; Gelly, J.C.; Swapna, L.S.; Offmann, B.; Cadet, F.; Bornot, A.; Tyagi, M.; Valadié, H. A short survey on protein blocks. *Biophys Rev* **2010**, *2*, 137-145.
5. Faure, G.; Joseph, A.P.; Craveur, P.; Narwani, T.J.; Srinivasan, N.; Gelly, J.C.; Rebehmed, J.; de Brevern, A.G. Ipbavizu: A pymol plugin for an efficient 3d protein structure superimposition approach. *Source code for biology and medicine* **2019**, *14*, 5.
6. Joseph, A.P.; Srinivasan, N.; de Brevern, A.G. Improvement of protein structure comparison using a structural alphabet. *Biochimie* **2011**, *93*, 1434-1445.
7. Léonard, S.; Joseph, A.P.; Srinivasan, N.; Gelly, J.C.; de Brevern, A.G. Mulpba: An efficient multiple protein structure alignment method based on a structural alphabet. *Journal of biomolecular structure & dynamics* **2014**, *32*, 661-668.
8. Craveur, P.; Joseph, A.P.; Esque, J.; Narwani, T.J.; Noël, F.; Shinada, N.; Goguet, M.; Leonard, S.; Poulain, P.; Bertrand, O.*, et al.* Protein flexibility in the light of structural alphabets. *Frontiers in molecular biosciences* **2015**, *2*, 20.
9. de Brevern, A.G. Analysis of protein disorder predictions in the light of a protein structural alphabet. *Biomolecules* **2020**, *10*.
